# Clinical genetic testing outcome with multi-gene panel in Asian patients with multiple primary cancers

**DOI:** 10.18632/oncotarget.25769

**Published:** 2018-07-17

**Authors:** Gloria H.J. Chan, Pei Yi Ong, Jeffrey J.H. Low, Hwai Loong Kong, Samuel G.W. Ow, David S.P. Tan, Yi Wan Lim, Siew Eng Lim, Soo-Chin Lee

**Affiliations:** ^1^ Department of Haematology-Oncology, National University Cancer Institute, Singapore (NCIS), Singapore; ^2^ Department of Obstetrics and Gynaecology, National University Hospital, Singapore; ^3^ Cancer Science Institute, Singapore

**Keywords:** genetic testing, germ-line mutation, neoplasms, multiple primary/diagnosis

## Abstract

**Background:**

Developing multiple cancers is an indicator of underlying hereditary cancer predisposition, but there is a paucity of data regarding the clinical genetic testing outcomes of these patients.

**Methods:**

We compared cancer index patients with ≥2 primary malignancies versus 1 primary cancer who underwent clinical evaluation and testing with multi-gene panels comprising up to 49 genes from 1998-2016.

**Results:**

Among 1191 cancer index patients, 80.6%, 17.2%, and 2.2% respectively had 1, 2, and ≥3 primary malignancies. For patients with 2 primary cancers (n=205), the most common cancer pairs were bilateral breast (37.5%), breast-ovary (11.7%), endometrium-ovary (9.2%), colon-endometrium (3.9%) and colon-colon (3.4%). 42.3% patients underwent gene testing including 110/231 (47.6%) with multiple malignancies. Pathogenic variants were found more frequently in younger patients, in those with a family history of cancer related to the suspected syndrome, and a trend towards significance in those with multiple primary cancers (35.5% vs. 25.6%, p = 0.09). In patients with multiple cancers, pathogenic variants were most commonly identified in BRCA1 (38.5%), BRCA2 (17.9%), and the mismatch repair genes (20.5%), while 23.1% of pathogenic mutations were in other moderate- to high-penetrance cancer predisposition genes including APC, ATM, MUTYH, PALB2, RAD50 and TP53.

**Conclusion:**

Patients with multiple cancers were more likely to carry pathogenic mutations than those with single cancer. About three-quarters of deleterious mutations in patients with multiple primary cancers were in BRCA1/2 and the mismatch repair genes, but multi-gene panel testing facilitated the detection of mutations in another 6 genes and is warranted in this high-risk population.

## INTRODUCTION

Developing multiple primary cancers may be due to a common exposure (e.g., radiation, tobacco-smoking, papilloma virus or a susceptibility gene). International clinical practice guidelines advocate cancer genetic screening for patients with multiple primary cancers [1, 2]. However, there is still a paucity of data regarding the characteristics and clinical genetic testing outcome of patients with multiple primary cancers, particularly in Asia. Here, we describe the characteristics and genetic testing outcomes of patients with multiple versus single primary cancers who were evaluated in a cancer genetics clinic in a tertiary cancer center in Asia.

## RESULTS

### Patients’ characteristics

1191 cancer index patients seen at the NCIS cancer genetics clinic were included in this study (Table 1). 84.6% were female and 67.6% were Chinese. The most commonly suspected hereditary cancer syndromes were hereditary breast and ovarian cancer (n = 737; 61.8%) and Lynch syndrome (n = 311; 26.1%); 61% had a family history consistent with the suspected hereditary cancer syndrome. 960 (80.6%) patients have a single primary cancer and 231 (19.4%) patients have multiple primary cancers (range 2 - 5).

**Table 1:** Patient demographics (N=1191)

		Patients with single primary cancer (n=960) No. (%)	Patients with multiple primary cancers (n=231) No. (%)
Age at first cancer diagnosis (median, range; years)		42 (11-85)	46 (21-87)
Gender	Female	802 (83.5)	206 (89.1)
	Male	158 (16.5)	20 (10.9)
Race	Chinese	631 (65.7)	175 (75.8)
	Malay	89 (9.3)	20 (8.7)
	Indian	53 (5.6)	12 (5.2)
	Others	184 (19.5)	24 (10.4)
Family history of cancer	Consistent with suspected syndrome	596 (62.0)	131 (56.7)
	Any	733 (76.3)	179 (77.4)
Primary suspected hereditary cancer syndromes	Hereditary breast and ovarian cancer	613 (63.8)	124 (53.7)
	Lynch syndrome	239 (24.8)	77 (33.3)
	Li-Fraumeni syndrome	18 (1.8)	4 (1.7)
	Others^	90 (9.3)	26 (11.3)

^Others: Cowden Syndrome (n = 17), familial adenomatous polyposis (n = 17), hereditary diffuse gastric cancer (n = 12), Von-Hippel-Lindau Syndrome (n = 8), Multiple endocrine neoplasia (MEN) syndrome (n = 7), hereditary paraganglioma-pheochromocytoma syndrome (n = 1), Peutz-Jegher syndrome (n = 1), hereditary leiomyomatosis and renal cell cancer (n = 1).

**Figure 1:**
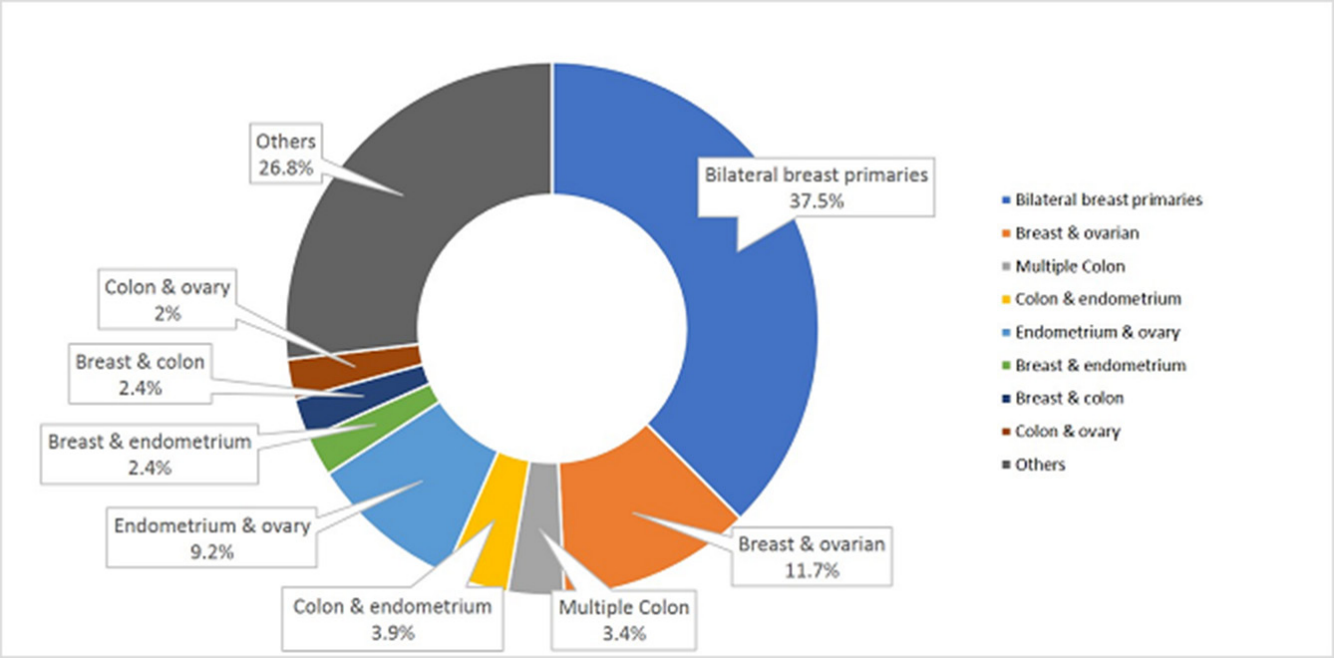
Distribution of multiple primary cancers in patients with 2 primary cancers (n = 205)

Amongst the 231 patients with multiple primary cancers, majority (205/231, 88.7%) had two primary malignancies (Figure 1). The most common cancer pairs were bilateral breasts (n = 77), breast and ovary (n = 24), endometrium and ovary (n = 19), colon and endometrium (n = 8), multiple colon (n = 7), breast and endometrium (n = 5), breast and colon (n = 5), colon and ovary (n = 5), and others (n = 55). Twenty-three patients (9.9%) had 3 cancers, with the most common being a combination of breast and ovarian cancers (n = 7), and multiple synchronous or metachronous colon primaries (n = 3). One patient had 5 primary cancers originating from 5 different organs (breast, lung, colon, skin, pancreas) (Table 2). The most common primary suspected cancer syndromes were hereditary breast and ovarian cancer syndrome (53.7%) and Lynch Syndrome (33.3%) in patients with multiple primary cancers.

**Table 2:** Patients with ≥3 primary malignancies

Patients who underwent genetic testing		
Site of primary malignancies (age at diagnosis)	Family history (age at diagnosis)	Genetic test outcome
Breast (50), parathyroid (60), endometrium (61)	Sister – breast (64) Niece – endometrium (NR)	RAD50 c.2165_2166INST (p.LYS722ASNfs^*^6)
Breast (50, 55), ovary (58)	Sister – nasopharyngeal (30) Maternal uncle – colon (50)	MUTYH c.934-2A>G (splice acceptor) in intron 10
Breast (40, 47), ovary (51)	Sister – ovary (48) Father – stomach (62) Paternal uncle – prostate (80’s) Paternal cousin – breast (50)	BRCA2: 9143delT
Breast (32, 34), ovary (45)	N.A.	VUS in DICER1, POLE, TP53 Initial BRCA1/2 negative
Colon (30, 46, 46)	Mother – endometrium (50) Maternal aunt – bladder (30) Maternal aunt – colon (40) Maternal cousin – colon, endometrium (30)	VUS in MLH1, MSH2
Colon (52), ovary (52), endometrium (52)	Mother – colon (66)	VUS in MLH1, MSH2
Colon (44, 45, 55)	Maternal aunt – endometrium (70)	MLH1 c.1731A>G
Endometrium (53), breast (57, 69)	N.A.	VUS In VHL
Liver (56), kidney (67), colon (73)	N.A.	49 gene panel negative
Lymphoma (63), colon (65), bladder (66)	N.A.	VUS in BRIP1, MSH6, PALLD
Ampullary (38), endometrium (48), colon (54)	Father – stomach (60)	MLH1 delK618
Ovary (50), breast (53), peritoneum (69)	Daughter – breast (38) Sister – breast (51) Maternal aunt – breast (30)	VUS in APC, MET, MSH6, PDGFR1, TSC2
Ovary (44), breast (48, 48)	N.A.	BRCA1 c.2726DUPA (p.Asn909Lysfs^*^6) in exon 10
Thyroid (58), endometrium (66), breast (67)	N.A.	Negative for PTEN
Breast (59), lung (68), colon (69), skin (71), pancreas (72)	Sister – colon (59) Mother – breast (75), colon (87)	TP53 c.733G>A (p.Gly245Ser) in exon 7

Patients who did not undergo genetic testing	
Site of primary malignancies (age at diagnosis)	Family history (age at diagnosis)
Breast (53), ovary (55), colon (57)	Father – stomach (63)
Breast (36, 52, 60)	Mother – breast (70) Paternal cousin – breast (50’s)
Breast (52, 52), ovary (52)	N.A.
Colon (71), skin (82, 83)	N.A.
Colon (39), endometrium (41) ovary (41)	N.A.
Colon (53), Breast (55, 64)	Father – liver (50)
Colon (49, 49, 49)	Paternal aunt – breast (60) Paternal grandfather – esophagus (70) Maternal grandmother (65)
Colon (53), Breast (55, 64)	Father – liver (50)
Colon (49, 49, 49)	Paternal aunt – breast (60) Paternal grandfather – esophagus (70) Maternal grandmother (65)
Parotid (39), lung (45), thyroid (48)	Mother – lymphoma (69) Father – lung (53)
Stomach (49), breast (53, 54)	N.A.
Colon (37), endometrium (38), thyroid (55), ovary (65)	N.A.
Colon (45), cervix (52), bladder (55), endometrium (57), colon (61)	N.A.

Compared to patients with single primary cancers, those with multiple primary cancers had a slightly older median age at first cancer diagnosis (46 vs. 42 years), were more likely to be Chinese (75.8% vs. 65.7%) and were less likely to have family history of cancer related to the primary suspected syndrome (56.7% vs. 62.0%).

### Genetic testing uptake and outcome

504/1191 patients (42.3%) eventually underwent germline genetic testing. Patients with multiple primary cancers were more likely to undergo testing than patients with 1 primary cancer (47.6% vs 41.0%, p=0.07), although the difference did not reach statistical significance. Approximately half the patients tested underwent multi-gene panel testing (51/110 [46.3%] versus 201/394 [51.9%] for patients with multiple versus single primary cancers). Overall, 27.7% (n=140) of patients tested were found to carry deleterious mutations, spanning 11 genes in patients with multiple primary cancers (n = 38) and 16 genes in those with single primary cancer (n = 102). Patients with multiple primary cancers were more likely to be diagnosed with deleterious mutations than those with single primary cancers (35.5% versus 25.6%, p = 0.09), although the difference did not reach statistical significance. Among patients who underwent genetic testing, those who had younger onset cancer (age of first cancer diagnosis < 45; p = 0.02) and those with a family history of cancer related to the primary suspected syndrome (p < 0.001) were more likely to carry a deleterious mutation. There was no significant correlation between identifying a pathogenic mutation and primary suspected syndrome or type of test used (Table 3).

**Table 3:** Factors that correlate with the identification of pathogenic mutations (n = 504)

All patients who underwent cancer genetic testing (n = 504)		No. of patients with pathogenic variants (%)	p-value
No. of cancers	Single primary cancer (n = 394)	102 (25.8)	0.09
	Multiple primary cancers (n = 110)	38 (34.5)	
Age at first cancer diagnosis	< 45 (n = 290)	89 (30.6)	0.02
	≥ 45 (n = 214)	51 (23.8)	
Family history of cancer related to suspected syndrome	Yes (n = 312)	108 (34.6)	<0.001
	No (n = 192)	32 (16.6)	
Type of test used	Targeted gene testing (n = 252)	68 (26.9)	0.76
	Cancer panel (n = 252)	72 (28.5)	
Primary suspected syndromes	BRCA (n = 346)	95 (27.4)	0.94
	Lynch (n = 118)	33 (27.9)	
	Others (n = 40)	12 (30.0)	

Patients with multiple primary cancers (n = 231)			
**No. of cancers**	2 primary cancers (n = 205)	31 (15.1)	0.20
	>2 primary cancers (n = 26)	7 (26.9)	

### Spectrum of deleterious mutations (Figure 2, Table 4 & Supplementary Table 1)

In patients with multiple primary cancers, pathogenic variants were most commonly identified in *BRCA1* (38.5%), *BRCA2* (17.9%), and the mismatch repair genes – *MLH1, MSH2, MSH6* (20.5%), while 23.1% of pathogenic mutations were in other moderate- to high-penetrance cancer predisposition genes. This distribution is similar to that observed in patients with one primary cancer, in whom pathogenic variants in *BRCA1* (35.0%), *BRCA2* (23.1%), and the mismatch repair genes (15.4%) accounted for almost three-quarters of deleterious mutations. Of note, the likelihood of identifying *TP53* deleterious mutations was similar in patients with multiple primary cancers compared to those with single primary cancer (2.7% vs 1.2%).

**Figure 2:**
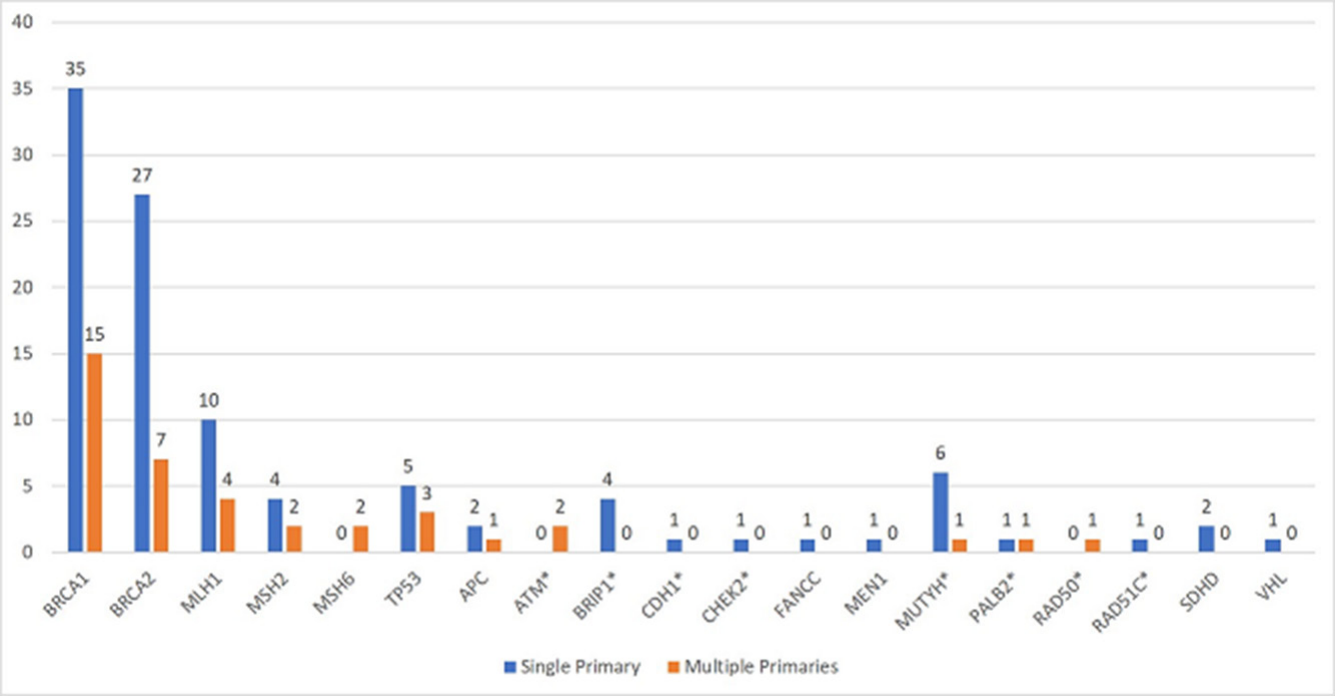
Pathogenic variants identified

**Table 4:** Deleterious mutations in patients with 2 primary cancers*

Gene	Mutation	Personal cancers (age at diagnosis)	Family history (age at diagnosis)
**APC**			
	APC c.4031C>G, p.(Ser1344^*^)	Thyroid (24) Desmoid tumour (32)	Brother – colon (32) Sister – colon (28) Sister – colon (40’s) Mother – colon (30’s)
**ATM**			
	ATM c. 785T>A (p.Leu262^*^) in exon 7ATM c.8494C>T (p.Arg2832Cys) in exon 58VUS in PMS2	Breast (64) Endometrium (64)	Brother, 2 maternal 1st cousins – leukaemia (all 50’s) Maternal uncle – liver (70) Maternal uncle – kidney (59) Maternal first cousin – lung (50’s)
	ATM c.1126_1127delGA	Colon (36) Breast (51)	Mother – breast (60) Maternal first cousin – breast (30’s) Maternal uncle – rectal (NR)^*^
**BRCA1**			
	BRCA1 2372delGT	Breast (38) Ovarian (39)	Maternal aunt – breast (60) Paternal cousin – breast (48) Paternal cousin – brain (60) Paternal cousin – colon (60) Paternal uncle – colon (73) Father – lung (NR)
	BRCA1 1966DelC (STOP625)	Breast (31) Breast (31)	Mother – ovarian (64) Maternal aunt – endometrium (40’s) Maternal grandmother – ovarian (40’s) Maternal cousin – breast (35)
	BRCA1 2845insA	Breast (42) Breast (46)	Mother – breast (60’s)
	BRCA1 c.3214DelC (p.Leu1072^*^) in exon 10	Breast (44) Ovarian (49)	Sister – breast (40’s) Sister – breast (40’s)
	BRCA1 c.4386del p.(Glu1462fs)	Breast (55) Ovarian (62)	Sister – ovarian (46) Sister – ovarian (54) Paternal uncle – prostate (70’s) Maternal cousin – colon (40)
	BRCA1 c.5467+1G>A	Breast (48) Breast (55)	Sister – breast (40) Paternal uncle – stomach (72) Paternal uncle – colon (NR)
	BRCA1 E879X	Breast (35) Breast (58)	Sister – breast (46) Father – liver (49)
	BRCA1 c.2276delA	Breast (58) Ovarian (58)	Sister – breast (39, 54), sarcoma (50)
	BRCA1 2276delA	Breast (39) Breast (50)	Sister – breast (40’s, 50’s)
	BRCA1 c.3916_3917DelT (p.Leu1306Aspfs^*^23) in exon 10	Ovarian (48) Breast (57)	Mother – ovarian (53) Sister – breast (27)
	BRCA1 c.2845insA	Rectovaginal (41) Breast (47)	Nil
	BRCA1 c.213-12A>G in intron 4	Breast (35) Breast (44)	Sister – breast (43) Mother – breast (50’s, 60’s)
	BRCA1 c.4065_4068DelTCAA (p.Asn1355Lysfs^*^) in exon 10	Breast (54) Breast (66)	Sister – breast (40) Niece – breast (31)
	BRCA1 Truncating mutation in exon 11B	Breast (36) Breast (38)	Nil
**BRCA2**			
	BRCA2 9189del4	Breast (44) Breast (53)	2 brothers – NPC (40’s, 40’s) Mother – breast (54), tongue (63)
	BRCA2 c.2095_2096delCA (p.Gln699Valfs^*^8) in exon 11	Breast (45) Ovarian (58)	Maternal uncle – liver (30’s)
	BRCA2 c.7878G>A	Breast (44) Breast (44)	Maternal aunt – cervix (65)
	BRCA2 c.2808_2811delACAA (p.Ala938ProfsX21)	Breast (40) Breast (44)	Mother – peritoneum (NR) Maternal aunt – breast (30’s) Maternal cousin – breast (41)
	BRCA2 1090delCCAAATG	Breast (35) Breast (44)	Sister – breast (35) Sister – ovarian (40) Mother – breast (38)
	BRCA2 c.9414_941delAT p.(Leu3138fs)	Breast (25) Breast (30)	Mother – breast (30) Maternal aunt) – breast (40)
**MLH1**			
	MLH1 2101C>A	Colon (54) Endometrium (55)	Brother – colon (43) Daughter – colon (31) Paternal cousin – bladder (34)
	MLH1 c.2041G>A (p.Ala681Thr) in exon 18	Breast (41) Colon (53)	Sister – colon (21) Father – head & neck (NR)
**MSH2**			
	MSH2 c.942+3A>T in intron 5	Ovarian (45) Endometrium (45)	Sister – endometrium (44) Sister – endometrium (41) Brother – colon (NR)
	MSH2 c.2210+1 G>A (splice donor) in intron 13	Endometrium (43) Ovarian (43)	Sister – endometrium (46)
**MSH6**			
	MSH6 c.3261delC (p.Phe1088Leufs^*^5) in exon 5	Breast (52) Endometrium (64)	Brother – bladder (56) Sister – breast (58)
**Gene**			
	MSH6 c.2230dupG (p.Glu744Glyfs^*^12) in exon 4	Endometrium (43) Ovarian (43)	Mother – tongue (76)
**PALB2**			
	PALB2 c.7G>T (p.Glu3^*^) in exon 1	Breast (30) Endometrium (40)	Nil
**TP53**			
	c.365_366del (p.Val122Aspfs^*^26)	Breast (32) Sarcoma (34)	Mother – lung (46) Maternal grandfather – prostate (70’s)
	c.817C>T (p.Arg273Cys)	Tongue (28) Breast (30)	Mother – breast (43) Maternal aunt – breast (38), lung (53)

^*^Note: mutations in patients with >2 cancers reported in Table 2; NR: Not recorded.

Of the 252 patients who underwent multi-gene panel testing, 26.3% of those with single primary cancer (n = 201) and 37.2% of those with multiple primary cancers (n = 51) were found to carry a deleterious mutation (p = 0.16). Deleterious mutations in the *BRCA1/2* genes were detected in 13% of each group, while mismatch repair gene mutations were detected in 2.4% and 9.8% of patients with single versus multiple primary cancers respectively. Multi-gene panel testing identified deleterious mutations in genes other than *BRCA1/2* or mismatch repair protein genes in 10.4% of patients with single primary cancers and in 13.7% of patients with multiple primary cancers. In those with multiple primary cancers, deleterious mutations were identified in *ATM, MUTYH, PALB2, RAD50* and *TP53.*

### Variants of uncertain significance (VUS) (Figures 3 and 4 & Supplementary Table 2)

33.9% (n = 171) of patients had at least one VUS identified and gene panel testing yielded more VUS results than targeted gene testing (41.4% vs 22.1%; p < 0.001). The overall incidence of VUS was similar in patients with multiple versus one primary cancers (32.7% vs 33.6%). A total of 275 VUS were identified in 43 genes amongst the 504 patients tested. The median number of VUS detected per gene was 4 (range 1-52). Of note, *BRCA1*, *BRCA2*, *MLH1*, and *MSH2* had the largest number of VUS detected (n=20, 21, 28, 52 respectively). No significant difference in VUS rates were detected between Chinese vs non-Chinese patients (35.3% vs. 31.2%; p = 0.37).

**Figure 3:**
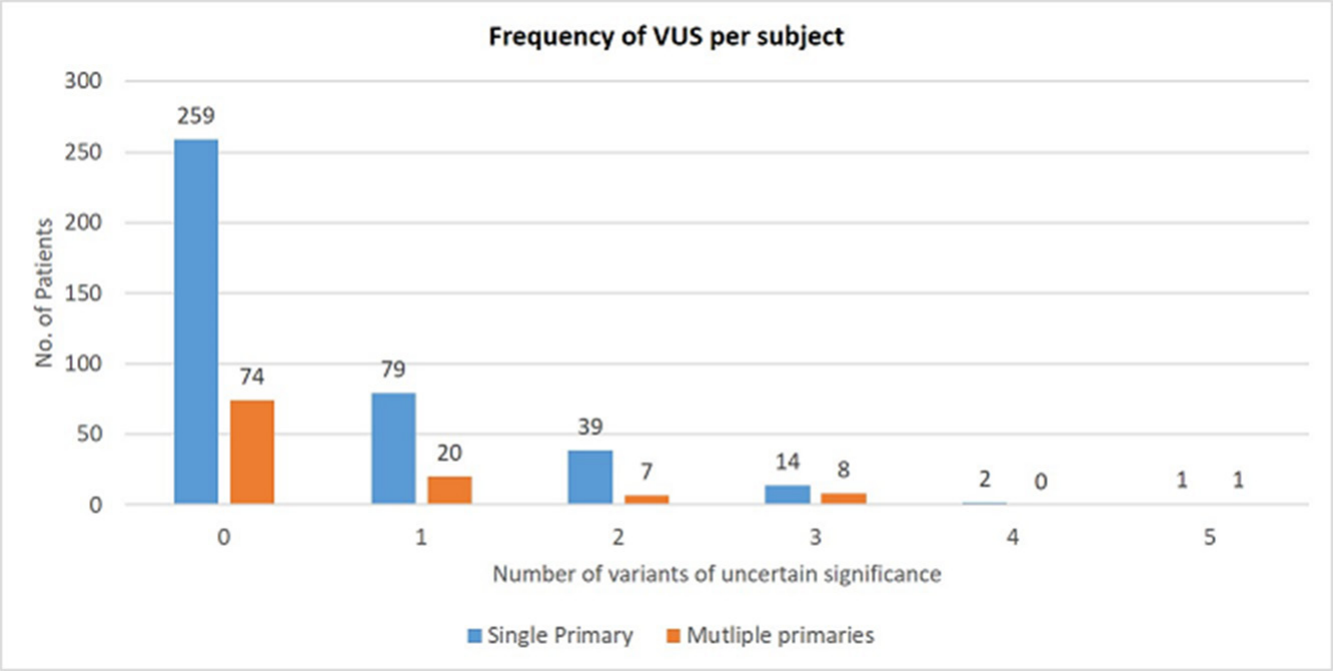
Variants of uncertain significance – Frequency per subject

**Figure 4:**
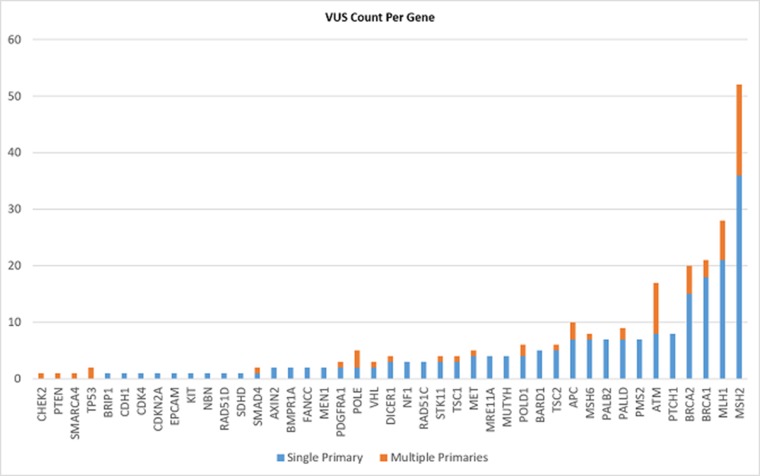
Variants of uncertain significance – VUS count per gene

## DISCUSSION

We describe a cohort of more than 1000 cancer patients suspected with hereditary cancer syndromes who were evaluated at a cancer genetics clinic in a tertiary cancer center in Asia. Patients with multiple primary cancers constitute 20% of patients in this cohort, of which the majority had two primary cancers and slightly more than 10% had 3 or more primary cancers. Half of these patients with multiple primary cancers underwent genetic testing and deleterious mutations were identified in one-third of patients, spanning 11 genes. To the best of our knowledge, this study is one of the largest on clinical cancer genetics testing outcomes in patients with multiple primary cancers in Asia, with multi-gene panel being used as the testing platform in about half the patients.

We found patients with multiple primary cancers to be more likely to carry deleterious mutations than those with single primary cancers (35.5% vs 25.6%), reflecting the results of other cohorts in the US and in Taiwan [3–5]. Of note, in the Taiwanese study, 50% of patients with a personal history of two cancers had mutations, compared to less than 25% in those with one cancer. Our results and that of others confirm that multiple primary cancers is associated with higher probability of an underlying hereditary predisposition, and that genetic counseling and genetic testing is warranted for these patients. However, despite the higher probability of carrying pathogenic mutations, patients with multiple primary cancers were not more likely than patients with single primary cancer to undergo testing in our study. Barriers to cancer genetic testing that have been reported in Asia include costs and misperceptions of testing outcomes [6]; addressing these barriers could potentially improve the uptake of testing among these high risk patients to optimize their management.

In our study, the most common cancer pairs were bilateral breasts, breast/ovary, endometrium/ovary, and colon/endometrium. These findings reflect the cancer patterns in Singapore, where colon cancer is the most common cancer among males, while breast, colon, ovarian, and endometrial cancers are among the top 5 cancers among females, in Singapore [7]. In comparison, in a European series of more than 200 patients with multiple primary cancers, the most common cancer pairs were breast/ovary and colon/endometrium. Similar to our study, half the patients in the European cohort underwent genetic testing, mostly targeted gene testing, and 40% were found to have a pathogenic variant [8]. Deleterious mutations were most frequently identified in mismatch repair genes (18.9%) and *BRCA1/2* genes (14.4%); other genes with deleterious mutations were *PTEN*, *RB1*, *APC* and *MUTYH* (homozygous), occurring in 6.3% of the cohort. In comparison, in our study, overall, 37% of patients with multiple primary cancers who underwent multi-gene panel testing were found to carry pathogenic mutations; 24% had mutations in the mismatch repair and *BRCA1/2* genes, while 13% had mutations in other cancer predisposition genes - more than two times than in the European study. The wider use of multi-gene panel testing in our cohort likely contributed to this finding. Furthermore, patients referred to the cancer genetics clinic represent a high-risk population that may have been further selected based on age and family history, in addition to personal history of multiple primary cancer. This ascertainment bias could be another reason why more than one-third of our patients with multiple primary cancers was found to carry deleterious mutations; this high incidence of deleterious mutations may not necessarily be representative of an unselected population with multiple primary cancers.

Several other groups outside Asia have reported their experience of panel testing in patients who initially test negative for *BRCA1/2*, identifying pathogenic variants in other moderate penetrance breast cancer genes in 7 – 10% of their cohorts, although most of these cohorts included only a minority of patients with multiple primary cancers [9–12]. Within Asia, a South Korean study of 235 patients at high-risk for hereditary breast cancer and were confirmed not to have a *BRCA1/2* mutation were tested with massively parallel sequencing, and 3.6% were found to have pathogenic germline mutations in *CHEK2*, *PALB2*, *MRE11* and *RAD50* [13]. Again, only a small minority of this cohort (5%) had multiple primary cancers. The wider availability and reducing cost of NGS-based multi-gene panel testing has led to increasing use of multi-gene panel testing for hereditary cancer syndrome in the clinic, in turn resulting in the rise in diagnosing cancer syndromes originating from other moderate to high penetrance cancer predisposition genes outside the mismatch repair and *BRCA1/2* genes. Multi-gene panel testing has the advantage of casting the net wider to improve the chance of diagnosing pathogenic mutations and could be advantageous in patients with multiple primary cancers and family histories suggestive of more than one hereditary cancer syndrome. For instance, in our cohort, a patient with a personal history of breast cancer at age 50, parathyroid malignancy at age 60 and endometrial cancer at age 61 and who has family history of breast, uterine and colon cancer had a primary suspected diagnosis of Lynch syndrome, while hereditary breast cancer syndrome and Cowden Syndrome were differential diagnoses. Multi-gene panel testing revealed no mutations in the mismatch repair, *BRCA1/2*, or *PTEN* genes but a deleterious frameshift mutation in *RAD50*, a moderate penetrance breast cancer gene. Nonetheless, although the panel used at our center comprises up to 49 genes, pathogenic variants have so far only been found in 19 genes, suggesting perhaps a more selective panel may be equally efficient.

Among patients who underwent multi-gene panel testing in our study, 7/252 patients (2.8%) were found to have a mono-allelic MUTYH pathogenic mutation, including 1 patient with multiple primary cancers. Mono-allelic MUTYH mutations have been reported in 1.4% of cancer-free controls, and 3.3% of colorectal cancer patients with family history. The frequency of mono-allelic MUTYH mutations in our patients with different primary cancers but who were all clinically suspected to have hereditary cancer predisposition is similar to what has been reported in the literature [14]. A large meta-analysis of genetic variants associated with colorectal cancer derived an aggregate relative risk of 1.17 (95% CI 1.01–1.34) for monoallelic mutations in MUTYH, less than the relative risk of having a first-degree relative with colorectal cancer (RR 2.25, 95% CI 2.00-2.53) [15]. The United States National Comprehensive Cancer Network’s (NCCN) recommendations for colorectal cancer screening for monoallelic MUTYH carriers are consistent with standard recommendations based on having a first-degree relative with colorectal cancer alone (i.e. screening colonoscopy every 5 years beginning at age 40, or 10 years prior to first-degree relative’s age at colorectal cancer diagnosis) [16]. Thus, the incidental identification of mono-allelic MUTYH mutation may be of limited clinical significance as it often does not alter medical management.

One inevitable by-product of multi-gene panel testing is the higher frequencies of VUS. It is well-established that the number of VUS directly correlates with the number of genes included on panels and the rates reported vary between 12 – 88% in the literature [17, 18]. Rates of VUS also vary amongst different ethnicities and are known to be highest among individuals with Asian ancestry due to low proportion of Asians included in reference databases [19], and our data reflects this. One third of our entire cohort had at least one VUS, although there was no significant difference in VUS rates between patients with multiple versus single primary cancers, or between different Asian ethnic groups. However, multi-gene panel testing was two times more likely to yield VUS compared to targeted gene testing. The identification of VUS can be associated with significant anxiety and may result in inappropriate implementation of management strategies that should be reserved for mutation carriers. This belies the importance of a cancer genetics clinic to provide adequate pre- and post-test counselling to this group of patients.

Patients with multiple primary cancers were more likely to carry pathogenic mutations in cancer predisposition genes than those with single primary cancer and constitute a high-risk group who should be referred for genetic counseling and testing. In our study, about one-third of Asian patients with multiple primary malignancies were diagnosed with pathogenic mutations, with three-quarters of deleterious mutations located in *BRCA1/2* and the mismatch repair genes. Multi-gene panel testing facilitated the detection of deleterious mutations in another 6 genes beyond *BRCA1/2* and the mismatch repair genes in more than 10% of patients with multiple primary cancers and may be considered in this high-risk population.

## MATERIALS AND METHODS

We studied all cancer index patients who have been referred to the cancer genetics clinic at the National University Cancer Institute, Singapore (NCIS) from 1998 to 2016. All cancer index patients had an *a priori* risk of ≥10% of having a hereditary cancer predisposition and received pre-test genetic counseling. The risk prediction models used for primary suspected syndrome were – Couch or PENN II models for hereditary breast and ovarian cancer syndrome; Amsterdam criteria, PREMM, or Bethesda for Lynch syndrome; and the Chompret’s or Eeles Criteria for Li-Fraumeni syndrome [20–26]. Clinical information including personal and three-generation family cancer histories, cancer type and histology were retrieved from the clinic databases. The study was approved by the Institution Ethics Review Board and informed consent was obtained from each participant.

Prior to 2014, clinical genetic testing comprised of targeted gene testing with Sanger sequencing and deletion/duplication analysis of suspected syndromes. After 2014, next-generation sequencing (NGS)-based multi-gene panel testing including up to 49 genes was performed, including full-gene sequencing and deletion/duplication analysis of the genes of interest. The 49 gene panel includes the following genes: *APC, ATM, AXIN2, BARD1, BMPR1A, BRCA1, BRCA2, BRIP1, CDH1, CDK4, CDKN2A, CHEK2, DICER1, EPCAM*, (deletion/duplication only), *FANCC, GREM1* (promoter region deletion/duplication only), KIT, MEN1, MET, MLH1, MRE11A, MSH2, MSH6, MUTYH, NBN, NF1, PALB2, PALLD, PDGFRA, PMS2, POLD1, POLE, PTCH1, PTEN, RAD50, RAD51C, RAD51D, RET, SDHA (sequence changes only), *SDHB, SDHC, SDHD, SMAD4, SMARCA4, STK11, TP53, TSC1, TSC2* and *VHL*.

Patients’ characteristics and genetic testing results were tabulated with descriptive statistics. Pearson’s chi-squared tests were performed to determine significant associations between categorical variables.

## SUPPLEMENTARY MATERIALS TABLES




