# Supplementary material for: Clinical genetic testing outcome with multi-gene panel in Asian patients with multiple primary cancers
Source: Oncotarget. 2018 Jul 17;9(55):30649–60. doi: 10.18632/oncotarget.25769 (PMC6078133; doi:10.18632/oncotarget.25769)
Supplement: Supplementary file 1 [file oncotarget-09-30649-s001.pdf]

Clinical genetic testing outcome with multi-gene panel in Asian patients with multiple primary cancers

SUPPLEMENTARY MATERIALS

Supplementary Table 1: Deleterious mutations in patients with single primary cancers

See Supplementry File 1

Supplementary Table 2: Variants of uncertain significance mutations identified

See Supplementry File 1
