# Supplementary material for: Clinical genetic testing outcome with multi-gene panel in Asian patients with multiple primary cancers
Source: Oncotarget. 2018 Jul 17;9(55):30649–60. doi: 10.18632/oncotarget.25769 (PMC6078133; doi:10.18632/oncotarget.25769)
Supplement: Supplementary file 2 [file oncotarget-09-30649-s002.docx]

**Supplementary Table 1: Deleterious mutations in patients with single primary cancers**

| No. | Mutation | Personal history (age at diagnosis) | Family history (age at diagnosis) |
| --- | --- | --- | --- |
| APC | | | |
| 1 | NR | Kidney (21) | Father – colon (58)  Paternal cousin – colon (30’s) |
| 2 | NR | Thyroid (19) | Father – colon (60’s)  Paternal uncle – colon (40’s)  Paternal cousin – hepatoblastoma (2) |
| BRCA1 | | | |
| 3 | BRCA1 c.3756_3759delGTCT | Breast (35) | Nil |
| 4 | BRCA1 2845insA | Breast (38) | Mother – ovarian (NR)  Maternal grandaunt – ovarian (NR) |
| 5 | BRCA1 c.5503C>T | Breast (34) | Paternal cousin – breast (34)  Paternal grandmother – breast (30’s) |
| 6 | BRCA1 c.4416_4417delTTinsG | Breast (30) | Mother – ovarian (31) |
| 7 | BRCA1 c.5137DELG p.(VAL1713*) | Breast (35) | Father – head and neck (55) |
| 8 | NR | Breast (38) | Mother – ovarian (61)  Maternal aunt – breast (50) |
| 9 | BRCA1 c.213-12A>G in intron 4 | Ovarian (42) | Father – esophageal (76) |
| 10 | BRCA1 K1601X | Breast (37) | Sister – breast (39)  Mother – breast (45) |
| 11 | BRCA1 c.2635G>T (p.E879X) | Breast (29) | Mother – breast (46)  Maternal aunt – breast (33, 58, 60)  Maternal grandfather – liver (49)  Paternal aunt – endometrium (30) |
| 12 | BRCA1 5382insC | Breast (33) | Mother – ovarian (53)  Maternal aunt – breast (33)  Maternal grandmother – breast (36) |
| 13 | BRCA1 c.5353C>T (p.Gln1785*) in exon 21 | Breast (49) | Sister – breast (37)  Mother – breast (44), ovarian (NR) |
| 14 | BRCA1 duplication of exon 12 (3 copies) | Ovarian (57) | Maternal aunt – ovarian (60’s)  Maternal aunt – breast (30’s) |
| 15 | BRCA1 c.2866_2870delTCTCA in exon 10 | Ovarian (39) | Father – prostate (60’s)  Paternal aunt – ovarian (40’s)  Paternal grandmother – NPC (NR) |
| 16 | BRCA1 c.981_982delAT (p.Cys328*) in exon 10 | Colon (53) | Sister – breast (39)  Mother – breast (55)  Maternal aunt – breast (60’s)  Maternal cousin – breast (55) |
| 17 | BRCA1 deletion of exon 3 | Breast (46) | Sister – ovarian (49)  Sister – breast (44)  Paternal uncle – skin (60)  Maternal aunt – endometrium (40’s) |
| 18 | BRCA1 c.68_69delAG (p.Glu23Valfs) in exon 2 | Breast (37) | Mother – breast (38)  Grandmother – ovarian (58) |
| 19 | BRCA1 deletion of exon 13-15 | Ovarian (41) | Maternal aunt – lung (59)  Paternal first cousin – leukemia (41) |
| Supplementary table 1 (continued) | | | |
| No. | **Mutation** | **Personal history (age at diagnosis)** | **Family history (age at diagnosis)** |
| 20 | BRCA1 c.4327C>T (p.Arg1443*) | Breast (33) | Sister – breast (34)  Mother – breast (51)  Father – esophageal (40’s)  Maternal aunt – breast (40)  Maternal grandmother – ovarian (58)  Maternal granduncle – stomach (70) |
| 21 | BRCA1 c.427G>T (p.Glu143*) in exon 6 | Cervix (33) | Maternal aunt – lung (NR) |
| 22 | BRCA1 c.68_69delAG (p.Glu23Valfs*17) in exon 2 | Breast (29) | Mother – ovarian (42)  Maternal grandmother – ovarian (40’s) |
| 23 | BRCA1 631insT  VUS in BRCA2 | Breast (43) | Sister – ovarian (40)  Nephew – leukemia (3)  Father – head and neck (63) |
| 24 | BRCA1 U14680.1: c.2071delA | Breast (41) | Sister – ovarian (45)  Father – colon (63)  Paternal grandmother – ovarian (52)  Paternal grandaunt – stomach  Maternal aunt – breast (65)  Maternal aunt – liver (50) |
| 25 | BRCA1 c.68_69DelAG (p.Glu23Valfs*17) in exon 2 | Ovarian (43) | Sister – ovarian (34)  Sister – ovarian (42)  Maternal aunt – ovarian (50’s) |
| 26 | BRCA1 c.2726dupA, p.(Asn909fs) in exon 11 | Ovarian (43) | Sister – ovarian (41)  Sister – ovarian (43)  Mother – peritoneum (60’s)  Maternal aunt – cervix (40’s) |
| 27 | BRCA1 Deletion in exon 13-15 | Ovarian (55) | Sister – breast (60)  Sister – breast (66)  Brother – colon (64)  Father – leukemia (70) |
| 28 | BRCA1 c.5074+1G>A (splice donor) in intron 16 | Ovarian (35) | Maternal grandmother – ovarian (60’s) |
| 29 | BRCA1 Deletion of exon 3 | Ovarian (49) | Sister – breast (46)  Sister – breast (44)  Paternal uncle – skin (60)  Maternal aunt – ovarian  Maternal aunt – endometrium (40)  Maternal first cousin – endometrium (NR) |
| 30 | BRCA1 3333delC in exon 11 | Breast (39) | Paternal aunt – breast (42) |
| 31 | BRCA1 c.4201C>T p.(Gln1401*) nonsense mutation | Ovarian (39) | Mother – colon (54)  Maternal aunt – ovarian (62)  Maternal aunt – ovarian (66)  Maternal aunt – breast (50)  Maternal aunt – breast (48)  Maternal uncle – prostate (70’s)  Maternal cousin – breast, breast (NR) |
| 32 | BRCA1 c.4201C>T p.(Gln1401*) in exon 13 | Ovarian (57) | Mother – colon (54) |
| Supplementary table 1 (continued) | | | |
| No. | **Mutation** | **Personal history (age at diagnosis)** | **Family history (age at diagnosis)** |
| 33 | BRCA1 c.3607C>T in exon 11 | Breast (44) | Mother – ovarian (47)  Maternal aunt – breast (60)  Maternal aunt – breast (60)  Maternal aunt – breast (32)  Maternal grandmother – cervical (64) |
| 34 | BRCA1 E879X  VUS in BRCA1: D345Y & BRCA2: R2842H | Breast (47) | Sister – breast (33, 59) |
| 35 | BRCA1 c.5525delT (p.Val1842Glufs*13) in exon 23  VUS in BMPR1A, NF1 | Ovarian (64) | Sister – oral (42)  Mother – colon (55) |
| 36 | BRCA1 1072delAins3  BRCA1 I89T (VUS) | Breast (38) | Brother – brain (27) |
| 37 | BRCA1 c.3661G>T (p.Glu1221*) in exon 10  VUS in MET and POLE | Ovarian (54) | Sister – ovarian (30’s)  Sister – breast (30’s)  Mother – pancreas (71)  Maternal cousin – nasopharynx (NR)  Paternal uncle – prostate (NR) |
| BRCA2 | | | |
| 38 | BRCA2 c.5656C>T (p.Gln1886*) in exon 11 | Ovarian (41) | Sister – breast (43)  Maternal first cousin – leukemia (20’s)  Maternal cousin – breast (30)  Paternal grandfather – breast (60’s) |
| 39 | BRCA2 c.774_775delAA (p.Glu260Serfs*15) in exon 9  MUTYH c.934-2A>G (splice acceptor) in intron 10 | Breast (37) | Maternal aunt – DCIS (50’s)  Paternal grandmother – throat (70’s)  Paternal second cousin – breast  Paternal second cousin – breast |
| 40 | BRCA2 c.9027T>G (p.Tyr3009*) in exon 23 | Breast (43) | Mother – Peritoneal (70) |
| 41 | BRCA2 deletion exon 17-18 | Urothelial (49) | Father – palate (82)  Paternal uncle – pancreas (70’s)  Paternal aunt – throat (50’s) |
| 42 | BRCA2 c.2442delC (p.Met815FS) | Breast (32) | Nil |
| 43 | BRCA2 duplication exon 4-11 | Breast (36) | Paternal uncle – stomach (60)  Paternal cousin – ovarian (45)  Paternal cousin – breast (1)  Paternal cousin – leukemia (27) |
| 44 | BRCA2 c.771_775delTCAAA (p.Asn257LYSfs*17) in exon 9 | Breast (35) | Sister – breast (53)  Sister – breast (44)  Mother – breast (48)  Father – lung (NR) |
| 45 | BRCA2 2699del6 | Male breast (54) | Grandmother – breast (60) |
| 46 | BRCA2 c.5351delA, p.(Asn1784fs) | Ovarian (43) | Sister – ovarian (38)  Mother – stomach (67)  Maternal uncle – liver (NR) |
| Supplementary table 1 (continued) | | | |
| No. | **Mutation** | **Personal history (age at diagnosis)** | **Family history (age at diagnosis)** |
| 47 | BRCA2 c.9376C>T (p.Gln3126*) | Breast (38) | Sister – ovarian (53)  Mother – brain (71)  Paternal aunt – breast (50’s)  Maternal cousin – pancreas (46)  Maternal cousin - cholangiocarcinoma (44)  Father – lung (72) |
| 48 | BRCA2 c.9376C>T (p.Gln3126*) in exon 25 | Ovarian (53) | Sister – breast (38)  Mother – brain (72)  Maternal cousin – pancreas (45)  Maternal cousin – pancreas (40) |
| 49 | BRCA2 c.658_659delGT | Ovarian (61) | Maternal aunt – nerve tumor (61)  Maternal grandmother – ovarian (60)  Father – lung (76)  Paternal aunt – breast (55)  Paternal cousin – melanoma (60)  Paternal cousin – melanoma (30’s) |
| 50 | BRCA2 c.262_263delCT (p.Leu88Alafs*12) in exon 3 | Ovarian (48) | Sister – breast (45)  Mother – endometrium (38), breast (62), lung (79) |
| 51 | BRCA2 c.5642_5645delAATC (p.Ser1882Lysfs*26) | Breast (44) | Father – pancreas (74)  Paternal aunt – leiomyosarcoma of stomach (59)  Paternal aunt – lung (60’s)  Paternal uncle – lung (60’s) |
| 52 | BRCA2 c.3109C>T (p.Gln1037*) in exon 11 | Breast (45) | Paternal aunt – breast (50’s)  Paternal grandmother – throat (70’s) |
| 53 | BRCA2 c.6275_6276delTT (p.Leu2092Profs*7) in exon 11 | Breast (41) | Father – prostate (67)  Paternal uncle – lung (60)  Paternal grandmother – ovarian (42) |
| 54 | BRCA2 c.5645C>A (p.Ser1882*) in exon 11  RET c.2410G>A (p.Val804Met) in exon 14 | Breast (49) | Mother - blood cancer (48)  Maternal aunt – Breast (50’s)  Maternal aunt – ovarian (40’s) |
| 55 | BRCA2 c.8585_8586delTA (p.Leu2862Argfs*6) | Ovarian (67) | Father - lung (NR) |
| 56 | BRCA2 2001del4 | Breast (36) | Nil |
| 57 | BRCA2 c.581G>A, p.(Trp194*) nonsense mutation  VUS in BRCA1 | Breast (64 – male) | Sister – throat (40) |
| 58 | BRCA2 c.8585_8586delTA (p.Leu2862Argfs*6) | Breast (44) | Sister – colon (38)  Other – ovarian (50’s)  Father – lung (65) |
| 59 | BRCA2 c.9097dupA, p.(Thr3033fs) | Breast (51) | Brother – colon (62), renal (63)  Sister – colon (61)  Sister – breast (40, 44)  Paternal uncle – gall bladder (51), lung (62) |
| Supplementary table 1 (continued) | | | |
| No. | **Mutation** | **Personal history (age at diagnosis)** | **Family history (age at diagnosis)** |
| 60 | BRCA2 Y2222X | Breast (39) | Sister – breast (39)  Mother – ovarian (41)  Brother – leukemia (33)  Maternal uncle – brain (40’s) |
| 61 | BRCA2 c.2808_2811delACAA | Breast (33) | Maternal cousin – breast (37)  Paternal grandmother – breast (50) |
| 62 | BRCA2 c.3865_3868delAAAT (p.Lys1289Alafs*3) in exon 11  VUS in MLH1 | Ovarian (72) | Son – breast (50’s)  Half-sister – stomach (60’s) |
| 63 | BRCA2 c.3109C>T (p.GLN1037*) in 11 VUS in TSC2 | Ovarian (46) | Mother – breast (56), lymphoma (60) |
| BRIP1 | | | |
| 64 | BRIP1 c.1343G>A (p.Trp448*) | Bladder (65) | Son – colon (39) |
| 65 | BRIP1 c.1343G>A (P.Trp448*) in exon 10 | Colon (39) | Father – bladder (65) |
| 66 | BRIP1 c.2947dupA (p.ILE983ANSfs*19) in exon 20  VUS in ATM, MSH6, PTCH1 | Breast (58) | Sister – brain (39) |
| 67 | BRIP1 c.505_506insAluY (p.Gln169Argfs*32) in exon 5  VUS in PALLD | Ovarian (62) | Nil |
| CDH1 | | | |
| 68 | CDH1 c.187C>T (p.Arg63*) in exon 3 | Pancreas (57) | Nephew – leukemia (5)  Maternal aunt – breast (55) |
| CHEK2 | | | |
| 69 | CHEK2 c.277delT (p.Trp93Glyfs*17) in exon 2 | Breast (34) | Mother – leukemia (57)  Gather – plasmacytoma (61)  Maternal grandmother – breast (50’s) |
| FANCC | | | |
| 70 | FANCC c.339G>A (p.TRP113*) in exon 4 | Breast (46) | Paternal first cousin – breast (29) |
| MEN1 | | | |
| 71 | MEN1 c.675_676delA | Pancreatic neuroendocrine tumor (25) | Nil |
| MLH1 | | | |
| 72 | MLH1 2108del10 | Colon (24) | Paternal aunt – stomach (45)  Paternal grandfather – leukemia (70), throat (70) |
| 73 | MLH1 IVS9+1G>A | Colon (32) | Father – lung (48)  Paternal uncle – colon (50’s)  Paternal aunt – colon (60;s)  Paternal cousin – breast (30’s)  Maternal aunt – skin (72)  Maternal aunt – cervix (34)  Maternal aunt – breast (42), gallbladder (43), pancreas (43)  Maternal aunt – breast (38), uterus (43) |
| Supplementary table 1 (continued) | | | |
| No. | **Mutation** | **Personal history (age at diagnosis)** | **Family history (age at diagnosis)** |
| 74 | MLH1 c.1072_1078del(p.Glu358Leufs*7) in exon 12 | Endometrium (48) | Sister – colon (37)  Brother – colon (48) |
| 75 | MLH1 c.704_723del(p.Lys236Glufs*64) in exon 9  MUTYH: c.934-2A>G (splice acceptor) in intron 10 | Colon (30) | Mother – colon (54)  Maternal grandfather – colon (75) |
| 76 | MLH1 c.790+1G>A (splice donor) in intron 9 | Endometrium (40) | Father – larynx (39), colon (42) |
| 77 | MLH1 c.298C>T | Colon (41) | Sister – endometrium (48)  Sister – colon (42)  Maternal uncle – colon (60)  Maternal aunt – colon (42), cervix (42)  Maternal aunt – colon (47), cervix (47)  Maternal aunt – colon (68)  Maternal aunt – cervix (47)  Maternal cousin – colon (36) |
| 78 | MLH1 c.1912G>T (p.Gly638*) in exon 17 | Ovarian (38) | Father – colon (60’s)  Paternal uncle – colon (30’s) |
| 79 | MLH1 c.1946delC | Colon (43) | Sister – ovarian (36)  Father – colon (51), colon (71)  Paternal uncle – colon (60’s)  Paternal cousin – colon (50’s)  Paternal cousin – colon (52), ovarian (52) |
| 80 | MLH1 c.1946delC  VUS MLH1 - 94G>A  VUS MSH2 -118C>T | Breast (45) | Sister – ovarian (36)  Brother – colon (43)  Father – colon (51), colon (71)  Paternal uncle – colon (60’s)  Paternal cousin – colon (50’s)  Paternal cousin – colon (52), ovarian (52) |
| 81 | MLH1 1151T>A | Colon (48) | Mother – colon (50;s)  Maternal aunt – colon (70’s)  Maternal grandfather – neck (70’s) |
| MSH2 | | | |
| 82 | MSH2 c.2551C>A | Ureter (54) | Brother – stomach (32)  Sister – breast (47)  Sister – breast (50) |
| 83 | MSH2 c.301G>T (p.Glu101*) in exon 2 | Colon (46) | Father – stomach (40), colon (67)  Paternal grandmother – endometrium (60’s) |
| 84 | MSH2 1168C>T missense mutation | Colon (47) | Mother – colon (43) |
| 85 | MSH2 c.1163dupA | Colon (59) | Brother – brain (50’s)  Father – colon (60)  Nephew – brain (38) |
| MUTYH | | | |
| 86 | MUTYH c.934-2A>G (splice acceptor) in intron 10 | Ovarian (67) |  |
| Supplementary table 1 (continued) | | | |
| No. | **Mutation** | **Personal history (age at diagnosis)** | **Family history (age at diagnosis)** |
| 87 | MUTYH c.934-2A>G (splice acceptor) in intron 10 | Gallbladder (40) | Father – liver (60)  Maternal aunt – ovarian (60) |
| 88 | MUTYH c.934-2A>G (splice acceptor) in intron 10 | Breast (40) | Paternal uncle – bladder (70’s) prostate (70’s)  Paternal uncle – lung (60)  Paternal aunt – endometrium (60)  Paternal aunt – breast (50’s)  Paternal aunt – breast (50) |
| 89 | MUTYH c.1240C>T (p.Gln414*) in exon 13 | Colon (57) | >100 Polyps |
| 90 | MUTYH c.934-2A>G (splice acceptor) in intron 10 | Breast (32) | Sister – breast (46)  Maternal uncle – pancreas (68) |
| 91 | MUTYH c.934-2A>G (splice acceptor) in intron 10  VUS in KIT | Thyroid (42) | Maternal uncle – testicular (60)  Maternal uncle – lung (60) |
| PALB2 | | | |
| 92 | PALB2 deletion exon 12-13 | Breast (28) | Brother – kidney (50)  Maternal aunt – colon (79)  Maternal aunt – breast |
| RAD51C | | | |
| 93 | RAD51C c.905-2A>C (splice acceptor) in intron 6 | Ovarian (53) | Mother – lung (85) |
| SDHD | | | |
| 94 | SDHD c.3G>C, p.(Met1) | Carotid paraganglioma (25) | Paternal first cousin – stomach (30) |
| 95 | SDHD c.3G>C p.(Met1) | Carotid paraganglioma (39) | Sister – carotid paraganglioma (36)  Brother – carotid paraganglioma (24) |
| TP53 | | | |
| 96 | TP53 c.541C>T (p.Arg181Cys) | Breast (43) | Paternal uncle – small bowel (70)  Paternal uncle – nose (50)  Paternal first cousin – breast (30) |
| 97 | TP53 c.541C>T (p.Arg181Cys) | Adrenal (26) | Sister – brain (3)  Mother – breast (38), lung (53)  Maternal aunt – breast (40)  Paternal uncle – liver (60)  Paternal grandfather – liver (70) |
| 98 | TP53 c.743G>A (p.Arg248Gln) | Breast (24) | Brother – adrenal (10) |
| 99 | TP53 c.524G>A (p.Arg175His) in exon 5; possibly mosaic | Ovarian (79) | Nil |
| 100 | TP53 c.716delA (p.Asn239THRfs*8) in exon 7  VUS in BARD1 | Colon (36) | Mother – breast (39), leukemia (51) |
| VHL | | | |
| 101 | VHL Deletion (entire coding sequence) | Cerebellar hemangioblastoma (42) | Brother – cerebellar hemangioblastoma (50) |

NR: Not recorded

**Supplementary table 2: VUS mutations identified**

| Mutation | Personal cancer (age at diagnosis) | Family history (age at diagnosis) |
| --- | --- | --- |
| APC |  |  |
| APC c.6364G>A (p.Ala2122Thr) | Colon (60) | Sister – breast (60's)  Maternal first cousin –colon (NR)  Father – lung (NR) |
| APC c.385G>C (p.Glu129Gln) in exon 4 | Ovary (50)  Breast (53)  Peritoneum (69) | Daughter – breast (38)  Sister – breast (48)  Maternal aunt – breast (30) |
| APC c.385G>C (p.Glu129Gln) in exon 4 | Breast (37) | Maternal grandaunt – endometrium (30’s)  Maternal second cousin – endometrium (29) |
| APC c.385G>C (p.Glu129Gln) in exon 4 | Breast (36)  Breast (47) | Nil |
| APC c.1114A>G (p.Asn372Asp) in exon 10 | Breast (38) | Mother – lung (71)  Maternal uncle – lung (50's)  Maternal uncle – throat (50's)  Paternal uncle – liver (60's)  Paternal aunt – breast (48)  Maternal aunt – colon (60's) |
| APC c.6553A>G (p.Ser2185Gly) in exon 16 | Gallbladder (59)  Pancreas (66) | Sister – breast (66)  Brother – cholangiocarcinoma (63) |
| APC c.8353A>C (p.Asn2785His) in exon 16 | Breast (32) | Paternal grandfather– bladder (60’s) |
| APC c.3632T>G (p.Met1211Arg) in exon 16 | Breast (48) | Nil |
| APC c.8233C>T (p.Pro2745Ser) in exon 16 | Ovary (44) | Paternal aunt – breast (50’s)  Paternal aunt – endometrium (50’s) |
| APC c.7211T>A (p.Met2404Lys) in exon 16 | Breast (38) | Father – stomach (65)  Paternal first cousin –breast (51) |
| ATM |  |  |
| ATM c.3295G>A (p.Asp1099Asn) in exon 23 | Breast (38) | Father – stomach (65)  Paternal first cousin –breast (51) |
| ATM c.283C>A (p.Gln95Lys) in exon 4 ATM c.512 A>G (p.Tyr171Cys) in exon 6 | Breast (65)  Breast (71) | Sister – breast (40’s) Sister – lung (60’s)  Sister – colon (62)  Sister – kidney (50’s)  Father – pancreas (NR) |
| ATM c.4910A>G (p.Asp1637Gly) in exon 33 | Breast (58) | Sister – brain (39) Mother – endometrium (40's)  Maternal uncle – lung (NR)  Half-brother – eye (4) |
| ATM c.1511A>G (p.Asn504Ser) in exon 10  ATM c.2630G>C (p.Ser877Thr) in exon 17 | Breast (33)  Breast (45) | Mother – breast (60’s)  Maternal aunt – thyroid (40’s)  Maternal grandfather – liver (50’s) |
| ATM c.8279T>C (p.Leu2760Pro) in exon 57 | Ovary (49) | Mother's paternal first cousin – breast (50’s) |
| Supplementary table 2 (continued) |  |  |
| Mutation | **Personal cancer (age at diagnosis)** | **Family history (age at diagnosis)** |
| ATM c.1703G>A (p.Arg568Lys) in exon 11 | Cervix (46)  Breast (52) | Paternal aunt – liver (50’s)  Paternal aunt – ovary (67) Paternal aunt – stomach (40’s)  Paternal aunt – peritoneum (65)  Maternal first cousin– lymphoma (20s) |
| ATM c. 1229T>C (p.Val410Ala) | Breast (46) | Mother – breast (50’s) Sister – breast (43) Maternal grandfather– lung (80) Father – prostate (70’s) Paternal grandmother – breast (NR) |
| ATM c.7566A>G (silent) in exon 51 | Breast (41) | Sister – fallopian tube (51)  Maternal uncle – colon (59)  Maternal grandfather – leukemia (80’s) |
| ATM c.275A>C (p.Lys92Thr) in exon 4 | Breast (59) Endometrium (49) | Niece – breast (30’s)  Maternal first cousin – breast (30’s)  Father – stomach (60's) |
| ATM c.5189G>A (p.Arg1730Gln) in exon 35 | Ovary (53)  Endometrium (53) | Sister – Endometrium (55) Father – bladder (60’s)  Mother – breast (70’s) |
| ATM c.1351C>T (p.Arg451Cys) in exon 10 | Breast (24)  Breast (44) | Paternal second cousin – breast (65) Maternal second cousin – breast (75) |
| ATM c.2095G>C (p.Glu699Gln) in exon 13 | Endometrium (47)  Ovary (47) | Father – liver (75) |
| ATM c.1903C>T (p.His635Tyr) in exon 13 | Ovary (62) | Brother – throat (50’s) Mother – lung (51)  Maternal grandmother – breast (70’s) |
| ATM c.7390T>C (p.Cys2464Arg) in exon 50 | Breast (49) | Paternal grandfather – prostate (65)  Mother – leukemia (74) |
| ATM c.1157G>A (p.Arg386Lys) in exon 9 | Breast (49) | Nil |
| AXIN2 |  |  |
| AXIN2 c.1802G>C (p.Gly601Ala) in exon 7 | Breast (43) | Mother – breast (69)  Maternal aunt – breast (60’s)  Maternal uncle – colon (NR)  Maternal grandmother – colon (NR) |
| AXIN2 c.721T>G (p.Cys241Gly) in exon 2 | Ovary (59) | Father – colon (65) |
| BARD1 |  |  |
| BARD1 c.643A>T (p.Asn215Tyr) | Ovary (43) | Mother – breast (59)  Maternal aunt –ovary (50’s) |
| Supplementary table 2 (continued) |  |  |
| Mutation | **Personal cancer (age at diagnosis)** | **Family history (age at diagnosis)** |
| BARD1 c.1702G>C (p.Gly568Arg) in exon 8 | Breast (33) | Mother – ovary/colon (51) Paternal aunt – colon (60’s) |
| BARD1 c.1601C>T (p.Thr534Ile) in exon 7  BARD1 c.2191c>T (p.Arg731Cys) in exon 11 | Breast (38) | Father – stomach (65)  Paternal first cousin – breast (51) |
| BMPR1A |  |  |
| BMPR1A c.713G>A (p.Arg238Gln) in exon 9 | Ovary (52) | Father – bone (60’s) |
| BMPR1A c.563G>A (p.Arg188His) in exon 8 | Peritoneum (64) | Sister – oral (42)  Mother – colon/endometrium (55) |
| BMPR1A c.1473A>G (silent) in exon 12 | Ovary (43) | Father – lymphoma (74)  Paternal uncle – prostate (NR)  Maternal aunt – liver (51)  Maternal uncle – liver (46)  Maternal uncle – (46) |
| BMPR1A c.1568A>G (p.Lys523arg) in exon 13 | Ovary (37) | Father – stomach (76)  Maternal aunt – cervix (50) |
| BMPR1A: c.3039T>A (silent) in exon 20 | Breast (43) | Maternal grandmother – bladder (60) |
| BMPR1A c.1520A>G (p.Asn507Ser) in exon 13 | Breast (49) | Nil |
| BRCA1 |  |  |
| BRCA1 c.5165C>T (p.Ser1722Phe) in exon 18 BRCA1 c.824G>A (p.Gly275Asp) in exon 10 | Ovary (47) | Sister – breast (40)  Brother – lung (40’s)  Father – lung (60’s) |
| BRCA1 c.1036C>T | Breast (35) | Mother – breast (55)  Maternal aunt – breast (49) |
| BRCA1 D345Y | Breast (47) | Sister – breast (33, 59) |
| BRCA1 c.1243G>A (p.Val415Ile) in exon 10 | Ovary (59) | Brother – colon (60’s)  Maternal aunt – breast (50’s)  Maternal uncle – colon (50)  Maternal uncle – liver (NR)  Maternal first cousin – colon (30’s)  Father – stomach (61)  Paternal uncle – nose (60’s) |
| BRCA1 c.3649T>C (p.Ser1217Pro) in exon 10 | Colon (20) | Father – lung (47) Paternal grandfather – colon (NR) |
| BRCA1 I89T | Breast (38) | Brother – brain (27) |
| BRCA1 c.2566T>C | Ovary (57)  Breast (68) | Nil |
| BRCA1 c.1114G>A p.R332Q | Breast (83) | Daughter – breast (50) |
| BRCA1 Intron 8 (Int.8-11)delA | Ovary (21) | Maternal aunt – breast (49) |
| BRCA1 c. 3997C>G, A1293G | Breast (49) | Niece – breast (36)  Maternal aunt – breast (40's)  Paternal aunt – breast (70) |
| BRCA1 V271M, IVS10-8A>G | Breast (23) | Nil |
| BRCA1 IVS11-11T>C | Breast (32)  Breast (34) | Nil |
| BRCA1 Duplication exon 1 and exon 2 | Breast (32) | Nil |
| BRCA1 c.3858G>A | Breast (29) | Nil |
| BRCA1 c.795T>C in exon 11 | Ovary (36) | Maternal aunt – breast (45) |
| Supplementary table 2 (continued) |  |  |
| Mutation | **Personal cancer (age at diagnosis)** | **Family history (age at diagnosis)** |
| BRCA1 c.544T>A (p.Leu182Met) in exon 7 | Prostate (59) | Brother – prostate (63)  Brother – prostate (62)  Brother – prostate (55)  Brother – prostate (52)  Sister – peritoneum (54) |
| BRCA1 c.2387C>T (p.Thr796Ile) in exon 10 | Breast (51) | Mother – colon (75), leukemia (77)  Maternal uncle –bladder (75) |
| BRCA1 c.2286A>T (p.Arg762Ser) in exon 10 | Prostate (51) | Brother – prostate (60’s)  Brother – prostate (60’s), brain (75)  Sister – breast (60’s)  Niece – breast (35)  Father – prostate (60’s) |
| BRCA1 c.4357+5_4357+6delGT in intron 12 | Peritoneum (60) | Sister – breast (40’s), thyroid (50’s) |
| BRCA1 c.5107T>C (p.Tyr1703His) in exon 17 | Peritoneum (53) | Father – lymphoma (78) Paternal uncle – liver (50’s)  Paternal aunt – breast (50’s)  Paternal grandaunt – breast (60’s)  Paternal grandaunt – breast (60’s)  Maternal aunt – stomach (60’s)  Maternal grandaunt – stomach (60’s)  Maternal grandaunt – stomach (60’s)  Maternal grandaunt – colon (60’s) |
| BRCA2 |  |  |
| BRCA2 c.4504C>G (p.Gln1502Glu) in exon 11 | Breast (41)  Colon (53) | Sister – colon (21)  Father – oral (60) |
| BRCA2 c.3540G>C (p.Lys1180Asn) | Ovary (46) | Mother – breast (45) |
| BRCA2 c.6638C>T | Breast (46) | 2 Paternal aunts – breast (50’s) |
| BRCA2 c.440A>G | Breast (48) | 2 Paternal aunts – breast (50’s)  Paternal cousin – breast (30’s) |
| BRCA2 C315S | Breast (39) | Mother – breast (40, 60)  Maternal grandmother – breast (70) |
| BRCA2 R2108C | Breast (53)  Nose (53) | Sister – breast (52)  Mother – breast (47) |
| BRCA2 D1513E | Breast (30) | Paternal uncle – colon (50's)  Paternal first cousin –kidney (38) Paternal grandmother – breast (30's)  Maternal grandmother – thyroid (30's), breast (40’s) |
| BRCA2 M1149V | Breast (34) | Nil |
| BRCA2 A2351G | Breast (32) | Nil |
| BRCA2 N830D (2716A>G) | Breast (42) | Nil |
| Supplementary table 2 (continued) |  |  |
| Mutation | **Personal cancer (age at diagnosis)** | **Family history (age at diagnosis)** |
| BRCA2 L2368V  BRCA2 IVS22-5DEL4 | Breast (73) | Daughter – breast (39)  Sister – pancreas (50's) Sister – breast (36)  Niece – breast (46) Niece – lymphoma (NR) Mother– breast (46), pancreas (58)  Maternal second cousin – parotid (NR)  Maternal second cousin – breast (39)  Maternal second cousin – breast (50) |
| BRCA2 c.91T>G (p.Trp31Gly) | Breast (38) | Paternal aunt – stomach (58)  Paternal first cousin – breast (44)  Paternal uncle – throat (50)  Paternal uncle – liver (51)  Maternal grandfather – lung (73) |
| BRCA2 c.5986G>A (p.Ala1996Thr) | Breast (40) | Nil |
| BRCA2 c.2671G>C (p.Val891Leu) in exon 11 | Breast (52)  Colon (57) | Mother – endometrium (60's) |
| BRCA2 c.5839C>T (p.Pro1947Ser) in exon 11 | Adrenal (39) | Nil |
| BRCA2 c.10043A>G (p.Asn3348Ser) in exon 27 | Colon (50)  Brain (58) | Sister – breast (60)  Mother – bladder (70’s)  Paternal first cousin – brain (40’s) |
| BRCA2 c.2122T>A (p.Ser708Thr) in exon 11 | Colon (23)  Desmoid (25) | Father – colon (61) |
| BRCA2 c.7052C>G  BRCA2 c.8187G>T | Breast (35) | Mother – breast (55)  Maternal aunt – breast (49) |
| BRCA2 R2842H | Breast (47) | Sister – breast (33, 59) |
| BRIP1 |  |  |
| BRIP1 c.1274A>T (p.Tyr425Phe) in exon 11 | Breast (43) | Maternal grandmother – bladder (60) |
| BRIP1 c.2441G>A (p.Arg814His) in exon 17 | Lymphoma (63)  Colon (65)  Paraganglioma (66) | Nil |
| BRIP1 c.2324A>G (p.Asn775Ser) in exon 16 | Ovary (58) | Sister – neck (21)  Father – colon (60) Paternal first cousin – colon (80’s) |
| BRIP1 c.2542C>T (p.Arg848Cys) in exon 18 | Ovary (52) | Father – bone (60’s) |
| CDH1 |  |  |
| CDH1 c.724G>A (p.Val242Ile) in exon 6 | Breast (43) | Mother – breast (60) Maternal first cousin – breast (50)  Father – prostate (72)  Paternal aunt – breast (60’s)  Paternal aunt – breast (50’s)  Paternal aunt – breast (50’s)  Paternal grandmother – stomach (60’s) |
| Supplementary table 2 (continued) |  |  |
| Mutation | **Personal cancer (age at diagnosis)** | **Family history (age at diagnosis)** |
| CDK4 |  |  |
| CDK4 c.776C>T (p.Ser259Leu) in exon 7 | Ovary (68) | Brother – lung (60’s)  Father – liver (70’s)  Paternal aunt – breast (50’s) |
| CDKN2A |  |  |
| CDKN2A c.26T>A (p.Met9Lys) in exon 1 | Breast (34) | Paternal grandfather – throat (NR) |
| CHEK2 |  |  |
| CHEK2 c.1438G>A (p.Ala480Thr) in exon 13 | Breast (59)  Breast (61) | Nil |
| DICER1 |  |  |
| DICER1 c.3334A>G (p.Asn1112Asp) in exon 21 | Breast (32)  Breast (34)  Ovary (45) | Paternal aunt – kidney (NR) |
| DICER1 c.1109T>C (p.Leu370Pro) in exon 8 | Ovary (47) | Mother – colon (83) |
| DICER1 c.1420A>G (p.Ile474Val) in exon 9 | Breast (36) | Paternal aunt – endometrium (68) |
| DICER1 c.1013A>C (p.Glu338Ala) in exon 8 | Ovary (64) | Nil |
| EPCAM |  |  |
| EPCAM c.232C>G (p.Leu78Val) in exon 3 | Peritoneum (41) | Paternal first cousin – breast (40’s)  Mother – lung (59) |
| FANCC |  |  |
| FANCC c.1000C>T (p.Arg334Trp) in exon 11 | Breast (55) | Paternal aunt – esophagus (60) Daughter of paternal first cousin – breast (30) |
| FANCC c.345+6A>T (intronic) | Ovary (43) | Mother – breast (59)  Maternal aunt – ovary (50’s) |
| KIT |  |  |
| KIT duplication of entire seq; copy number=3 | Thyroid (42) | Maternal uncle – testes (60’s)  Maternal uncle – lung (60’s)  Father – colon (50’s)  Paternal aunt – breast (40’s)  Paternal aunt – colon (60)  Paternal first cousin –breast (40’s)  Paternal grandfather – colon (30’s) |
| MEN1 |  |  |
| MEN1 c.634A>G (p.Asn212Asp) in exon 3 | Endometrium (58) | Sister – nose (43)  Maternal uncle – throat (60’s) |
| MEN1: c.1535C>T (p.Ser512Leu) in exon 10 |  | Sister – breast (36) |
| MET |  |  |
| MET c.4198C>T (p.Arg1400*) in exon 21 | Breast (43) | Sister – breast (36) |
| MET c.3253C>A (p.Gln1085Lys) in exon 15 | Ovary (54) | Sister – ovary (30's)  Sister – breast (30's)  Mother – pancreas (71)  Maternal first cousin – nose (NR)  Paternal uncle – prostate (NR) |
| MET c.1484C>G (p.Thr495Arg) in exon 4 | Parathyroid (41) | Maternal first cousin –pancreas (NR) |
| MET c.1912A>G (p.Ile638Val) in exon 7 | Ovary (50)  Breast (53)  Peritoneum (69) | Daughter – breast (38)  Sister – breast (48)  Maternal aunt – breast (30) |
| Supplementary table 2 (continued) |  |  |
| Mutation | **Personal cancer (age at diagnosis)** | **Family history (age at diagnosis)** |
| MLH1 |  |  |
| MLH1 474+79A>G | Ovary (49) | Sister – stomach (NR)  Sister – cervix (44)  Mother – cervix (71)  Maternal granduncle – brain (87)  Maternal granduncle – prostate (70’s)  Maternal grandmother – colon (70’s) |
| MLH1 -93G>A | Colon (30)  Colon (46) | Mother – endometrium (NR)  Maternal aunt – colon (NR)  Niece – colon (30), endometrium (30) Nephew – colon (30) |
| MLH1 -93G>A | Colon (52)  Ovary (52)  Endometrium (52) | Mother – colon (66) |
| MLH1 -93G>A | Colon (71)  Stomach (71) | Niece – breast (35) |
| MLH1 -93G>A | Breast (68)  Breast (73) | Sister – breast (47)  Sister – endometrium (78)  3 brothers – stomach (70’s)  Brother – colon (70’s) |
| MLH1 -93G>A | Sebaceous eyelid (77) | Brother – stomach (51)  Brother – stomach (52)  Nephew –colon (44) |
| MLH1 -93G>A | Colon (70) | Father – lung (NR)  Paternal uncle – lung (NR)  Paternal grandfather – lung (NR) |
| MLH1 -93G>A | Colon (33) | Maternal aunt – colon (44) |
| MLH1 -93G>A | Colon (65) | Nil |
| MLH1 -93G>A | Colon (37) | Paternal uncle – stomach (70)  Maternal uncle – esophagus (60)  Maternal uncle – lung (60’s)  Maternal uncle – colon (50) |
| MLH1 -93G>A | Colon (34) | Nil |
| MLH1 -94G>A | Ovary (47)  Endometrium (47) | Nil |
| MLH1 -94G>A | Colon (24) | Father – lung (NR)  Paternal first cousin – lymphoma (NR)  Maternal grandfather – lung (60’s)  Maternal grandmother – leukemia (NR) |
| MLH1 -94G>A | Stomach (54)  Colon (68) | Father – colon (64) |
| MLH1 -94G>A | Colon (39) | Paternal uncle – colon (50’s) |
| Supplementary table 2 (continued) |  |  |
| Mutation | **Personal cancer (age at diagnosis)** | **Family history (age at diagnosis)** |
| MLH1 -94G>A | Colon (57) | Nil |
| MLH1 -94G>A | Colon (42) | Paternal aunt – liver (80) |
| MLH1 -94G>A | Ovary (46)  Colon (49) | 2 sisters – endometrium (NR) |
| MLH1 -94G>A | Colon (57) | Brother – colon (38)  Father – colon (82) Mother – colon (93) |
| MLH1 c.2051A>G (p.Tyr684Cys) in exon 18 | Peritoneum (72) | Half-sister – stomach (NR) Son – breast (50’s) |
| MLH1 c.1487C>G (p.Pro496Arg) in exon 13 | Lung (60) | Brother – leukemia (57)  Niece – pancreas (44)  Mother – stomach (60's)  Maternal uncle – lung (NR) |
| MLH1 c.644A>G (p.Asn215Ser) in exon 8 | Peritoneum (78) | Niece – throat (50’s) |
| MLH1 c.1558+5G>A | Ovary (49) | Sister – breast (43)  Brother – prostate (47)  Maternal uncle – prostate (60’s) |
| MLH1 c.320T>A (p. Ile107lys) | Colon (36) | 2 Maternal uncles – colon (50’s)  Maternal grandmother – ovary (60’s)  Paternal grandmother – stomach (60’s)  Paternal first cousin – breast (40’s) |
| MLH1 c.320T>A (p. Ile107Lys) | Colon (56) | Daughter – colon (38)  Brother – colon (50's) |
| MLH1 2101C>A | Colon (62) | 2 brothers – colon (60’s)  Brother – prostate (69)  Nephew – colon (25)  Father – colon (68)  Paternal aunt – colon (70)  Paternal aunt –breast (90)  Paternal cousin – colon (50’s) |
| MLH1 c.1521G>C (p.Leu507Phe) in exon 13 | Breast (48) | Nil |
| MRE11A |  |  |
| MRE11A c.1724G>A (p.Gly575Asp) in exon 15 | Ovary (68) | Father – lung (60)  Daughter of maternal first cousin – brain (5) |
| MRE11A c.689C>T (p.Pro230Leu) in exon 8 | Colon (55) | Father – liver (79), colon (79) Paternal uncle – colon (30’s) Paternal aunt – colon/endometrium (50’s)  Paternal first cousin – prostate (63)  Maternal uncle – colon (75)  Maternal first cousin – prostate (63) |
| MRE11A c.1810C>T (p.Arg604Cys) in exon 16 | Breast (54) | Sister – ovary (45) |
| MRE11A c.1724G>A (p.Gly575Asp) in exon 15 | Colon (19) | Maternal uncle – liver (54) |
| Supplementary table 2 (continued) |  |  |
| Mutation | **Personal cancer (age at diagnosis)** | **Family history (age at diagnosis)** |
| MSH2 -118C>T  MSH2 211+9C>G  MSH2 2006-6T>C | Breast (33) | Mother – colon (78)  Maternal uncle – colon (70’s)  Maternal grandmother – colon (60’s) |
| MSH2 -118C>T  MSH2 211+9C>G | Colon (29) | Mother – colon (55) |
| MSH2 -118C>T  MSH2 211+9C>G | Colon (37) | Maternal uncle – colon (34) |
| MSH2 -118C>T  MSH2 211+9C>G | Colon (37)  Ovary (39) | Nil |
| MSH2 -118C>T  MSH2 211+9C>G | Colon (67) | Nil |
| MSH2 211+9C>G  MSH2 2006-6T>C  MSH2 -118C>T | Colon (30) | Paternal aunt – colon (40’s) |
| MSH2 c.1759+3A>T (intronic) in intron 11 | Ovary (47)  Endometrium (47) | Sister – endometrium (52)  Mother – colon (65), pancreas (65), breast (65)  Maternal aunt – lymphoma (60)  Maternal uncle – pancreas (70's) |
| MSH2 c.128A>G (p.Tyr43Cys) in exon 1 | Breast (90) | Maternal grandmother – breast (90)  Father – lung (60) |
| MSH2 c.2064G>A (p. Met668Ile) | Breast (33) | Mother – lung (63)  Maternal grandmother – lung (50's)  Paternal grandfather – stomach (50’s) |
| MSH2 211+9C>G | Colon (29) | Nil |
| MSH2 211+9C>G | Colon (43) | Mother – head/neck (20’s)  Maternal aunt – head/neck (50’s)  Maternal grandmother – head/neck (50’s) |
| MSH2 -118C>T | Stomach (61)  Kidney (60’s) | Sister – ovary (52)  Sister – colon (41)  Paternal cousin – colon (52) |
| MSH2 211+9C>G  MSH2 1661+12A>G  MSH2 2006-6T>C  MSH2 -118C>T | Ovary (49) | Sister – stomach (NR) Sister – cervix (44) Mother – cervix (71)  Maternal granduncle – brain (87)  Maternal granduncle – prostate (70’s)  Maternal grandmother – colon (70’s) |
| MSH2 1661+12A>G  MSH2 -118C>T | Colon (30)  Colon (46) | Mother – endometrium (NR)  Maternal aunt – colon (NR)  Niece – colon (30), endometrium (30)  Nephew – colon (30) |
| Supplementary table 2 (continued) |  |  |
| Mutation | **Personal cancer (age at diagnosis)** | **Family history (age at diagnosis)** |
| MSH2 -118C>T  MSH2 2006-6T>C | Breast (68)  Breast (73) | Sister – breast (47)  Sister – endometrium (78)  3 brothers – stomach (70’s)  Brother – colon (70’s) |
| MSH2 -118C>T  MSH2 211+9C>G  MSH2 2006-6T>C | Sebaceous eyelid (77) | Brother – stomach (51)  Brother – stomach (52)  Nephew – colon (44) |
| MSH2 1661+12A>G  MSH2 2006-6T>C  MSH2 -118C>T | Colon (70) | Father – lung (NR)  Paternal uncle – lung (NR)  Paternal grandfather – lung (NR) |
| MSH2 -118C>T  MSH2 211+9C>G | Colon (33) | Maternal aunt – colon (44) |
| MSH2 -118C>T  MSH2 211+9C>G | Colon (65) | Nil |
| MSH2 -118C>T | Colon (37) | Paternal uncle – stomach (70)  Maternal uncle – esophagus (60)  Maternal uncle – lung (60’s)  Maternal uncle – colon (50) |
| MSH2 -118C>T | Colon (34) | Nil |
| MSH2 -118C>T | Ovary (47)  Endometrium (47) | Nil |
| MSH2 -118C>T  MSH2 211+9C>G | Colon (24) | Father – lung (NR) Paternal first cousin – lymphoma (NR)  Maternal grandfather – lung (60’s)  Maternal grandmother – leukemia (NR) |
| MSH2 -118C>T | Stomach (54)  Colon (68) | Father – colon (64) |
| MSH2 -118C>T | Colon (39) | Paternal uncle – colon (50’s) |
| MSH2 -118C>T | Colon (40) | Paternal grandfather – stomach (NR)  Paternal uncle – liver (NR)  Paternal grandfather – stomach (NR)  Maternal aunt – breast (50’s)  Maternal uncle – leukemia (30’s) |
| MSH2 -118C>T | Colon (57) | Nil |
| MSH2 -118C>T | Colon (42) | Paternal aunt – liver (80) |
| MSH2 -118C>T | Ovary (46)  Colon (49) | 2 sisters – endometrium (NR) |
| MSH2 -118C>T | Colon (57) | Brother – colon (38)  Father – colon (82)  Mother – colon (93) |
| MSH2 c.871C>G (p.Leu291val) in exon 5 | Ovary (47) | Sister – breast (40)  Brother – lung (40’s)  Father – lung (60’s) |
| MSH2 c.956A>T (p.Asp319Val) in exon 6 | Breast (43) | Sister – breast (36) |
| MSH2 c.1789G>A (p.Asp597Asn) in exon 12 | Breast (38) | Father – stomach (65)  Paternal first cousin – breast (51) |
| Supplementary table 2 (continued) |  |  |
| Mutation | **Personal cancer (age at diagnosis)** | **Family history (age at diagnosis)** |
| MSH6 c.3283C>T (p.Arg1095Cys)  MSH6 c.3117C>T (p.Asn1039Asn) | Endometrium (51) | Mother – endometrium (45)  Father – colon (65)  Paternal aunt – breast (50)  Paternal aunt – breast (44)  Paternal grandmother – lymphoma (70’s) |
| MSH6 c.3980A>G (p.Asn1327Ser) in exon 9 | Breast (58) | Nil |
| MSH6 c.3205G>C (p.Gly1069Arg) in exon 5 | Ovary (33) | Paternal aunt – colon (50’s)  Paternal grandmother – endometrium (NR) |
| MSH6 c.136G>C (p.Gly46Arg) in exon 1 | Breast (28) | Brother – lymphoma (18)  Father – lymphoma (62)  Paternal grandfather – prostate (80’s) |
| MSH6 c.2235T>G (p.Ile745Met) in exon 4 | Breast (58) | Sister – brain (39) Mother – endometrium (40's)  Maternal uncle – lung (NR)  Brother – eye (4) |
| MSH6 c.3762A>T (p.Glu1254Asp) in exon 8 | Lymphoma (63)  Colon (65)  Paraganglioma (66) | Nil |
| MSH6 c.865T>C (p.Phe289Leu) in exon 5 | Ovary (50)  Breast (53)  Peritoneum (69) | Daughter – breast (38)  Sister – breast (48)  Maternal aunt – breast (30) |
| MSH6 c.4002-8A>C | Ovary (49) | Sister – stomach (NR)  Sister – cervix (44)  Mother – cervix (71)  Maternal granduncle – brain (87)  Maternal granduncle – prostate (70’s)  Maternal grandmother – colon (70’s) |
| MUTYH |  |  |
| MUTYH c.608G>C (p.Arg203Pro) in exon 8 | Breast (81) | Brother – lymphoma (40’s) Niece – breast (40’s)  Maternal aunt – endometrium (50’s)  Paternal aunt – colon (60’s)  Paternal aunt – breast (50’s) |
| MUTYH c.481G>C (p.Asp161His) in exon 6 | Prostate (53) | Father – prostate (75) |
| MUTYH c.934-2A>G (splice acceptor) in intron 10 | Breast (39) | Niece – kidney (3 months)  Paternal aunt – breast (40’s) |
| MUTYH c.1498_1502invCAACA (p.GlnGln500CysTrp) in exon 15 | Breast (48) | Nil |
| NF1 |  |  |
| NF1 c.2653G>A (p.Gly885Arg) in exon 21 | Sarcoma (60) | Father – esophagus (51) |
| NF1 c.5063T>G (p.Ile1688Arg) in exon 36 | Prostate (58) | Mother – breast (80’s) |
| NF1 c.3970A>G (p.Thr1324Ala) in exon 29 | Peritoneum (64) | Sister – oral (42)  Mother –colon/endometrium (55) |
| Supplementary table 2 (continued) |  |  |
| Mutation | **Personal cancer (age at diagnosis)** | **Family history (age at diagnosis)** |
| PALB2 c.380A>G (p.His127Arg) in exon 4 | Ovary (56) | Nil |
| PALB2 c.1054G>C (p.Glu352Gln) in exon 4 | Breast (33) | Nil |
| PALB2 c.149A>C (p.Lys50Thr) in exon 3 | Breast (25) | Nil |
| PALB2 c.3059A>G (p.Gln1020Arg) in exon 10 | Endometrium (46) | Mother – endometrium (77) |
| PALB2 c.1379A>G (p.Gln460Arg) in exon 4 | Breast (43) | Mother – breast (69)  Maternal aunt – breast (60’s)  Maternal uncle – colon (NR)  Maternal grandmother – colon (NR) |
| PALB2 c.2289G>C (p. Leu763Phe) in exon 5 | Peritoneum (41) | Paternal first cousin – breast (40’s)  Mother – lung (59) |
| PALB2 c.1097A>G (p.Asn366Ser) in exon 4 | Ovary (68) | Father – lung (60)  Daughter of maternal first cousin – brain (5) |
| PALLD |  |  |
| PALLD c.121C>G (p.Pro41Ala) in exon 2 | Ovary (62) | Nil |
| PALLD c.121C>G (p.Pro41Ala) in exon 2 | Thyroid (40’s)  Colon (60) | Sister – thyroid (50’s) Sister – thyroid (40’s)  Paternal first cousin – colon (50’s)  Son of paternal first cousin – colon (41)  Paternal first cousin –endometrium/ovary (50’s)  Paternal second cousin – colon (60’s)  Son of paternal second cousin – colon (30)  Maternal first cousin –colon (40’s) |
| PALLD c.121C>G (p.Pro41Ala) in exon 2 | Breast (55) | Mother – cervix/endometrium (30’s)  Maternal first cousin –ovarian/endometrium (40’s)  Paternal aunt – stomach (50’s)  Paternal second aunt – breast (60’s) |
| PALLD c.121C>G (p.Pro41Ala) in exon 2 | Colon (55) | Father – liver (79), colon (79) Paternal uncle – colon (30’s) Paternal aunt – colon (50’s)  Paternal first cousin – prostate (63)  Maternal uncle – colon (75)  Maternal first cousin – prostate (63) |
| PALLD c.736C>G (p.Gln246Glu) in exon 4 | Breast (28) | Brother – lymphoma (18)  Father – lymphoma (62)  Paternal grandfather –prostate (80’s) |
| PALLD c.121C>G (p.Pro41Ala) in exon 2 | Sarcoma (60) | Father – esophagus (51) |
| Supplementary table 2 (continued) |  |  |
| Mutation | **Personal cancer (age at diagnosis)** | **Family history (age at diagnosis)** |
| PALLD c.683C>T (p.Ala228Val) in exon 4 | Lymphoma (63)  Colon (65)  Paraganglioma (66) | Nil |
| PDGFRA |  |  |
| PDGFRA c.2768G>C (p.Ser923Thr) in exon 20 | Colon (61) | Brother – nose (49)  Mother – thyroid (70)  Maternal aunt – breast (70’s)  Maternal uncle – pancreas (70’s)  Maternal aunt – throat (70’s)  Maternal first cousin – lung (43)  Maternal first cousin – colon (40’s)  Maternal first cousin – stomach (50’s)  Maternal first cousin – liver (30’s)  Paternal first cousin – breast (50’s)  Paternal first cousin – brain (50’s)  Paternal first cousin – prostate (65) |
| PDGFRA c.452G>A (p.Arg151His) in exon 4 | Ovary (43) | Father – lymphoma (74)  Paternal uncle – prostate (NR)  Maternal aunt – liver (51)  Maternal uncle – liver (46)  Maternal uncle – (46) |
| PDGFRA c.2942G>A (p.Arg981His) in exon 22 | Ovary (50)  Breast (53)  Peritoneum (69) | Daughter – breast (38)  Sister – breast (48)  Maternal aunt – breast (30) |
| PMS2 |  |  |
| PMS2 c.1688_1689delGAinsAG (p.Arg563Gln) in exon 11  PMS2 c.1099G>A (p.Val367Ile) in exon 10 | Breast (49) | Nil |
| PMS2 c.58C>T (p.Arg20Trp) in exon 2 | Breast (33) | Nil |
| PMS2 c.2350G>A (p.Asp784Asn) in exon 14 | Ovary (64) | Sister – breast (29)  Sister – breast (53)  Mother – breast (80)  Maternal grandmother – breast (40’s)  Father – liver (70’s) |
| PMS2 c.166C>G (p.Leu56Val) in exon 3 |  | Sister – nose (36) Paternal uncle – lung (54) |
| PMS2 c.2149G>A (p.Val717Met) in exon 12 | Breast (34) | Nil |
| PMS2 c.883C>T (p.Arg295Trp) in exon 8 | Breast (33) | Nil |
| POLD1 |  |  |
| POLD1 c.49C>T (p.Arg17Trp) in exon 2 | Breast (43) | Father – liver (40's) |
| POLD1 c.2327G>A (p.Arg776Gln) in exon 19 | Ovary (53) | Paternal grandmother – endometrium (30’s) |
| POLE |  |  |
| POLE c.32c>T (p.Ala11Val) in exon 1 | Ovary (26) | Sister – breast (40's)  Brother – throat (50's) |
| POLE c.4855T>C (p.Tyr1619His) in exon 37 | Breast (40)  Breast (46) | Maternal first cousin – breast (56) Maternal first cousin – cervix (NR) |
| Supplementary table 2 (continued) |  |  |
| Mutation | **Personal cancer (age at diagnosis)** | **Family history (age at diagnosis)** |
| POLE c.4490A>G (p.Lys1497Arg) in exon 35 | Breast (41)  Colon (53) | Sister – colon (21)  Father – oral (60) |
| POLE c.2469-10G>A (intronic) in intron 21 | Breast (32)  Breast (34)  Ovary (45) | Paternal aunt – kidney (NR) |
| POLE c.5633G>A (p.Arg1878His) in exon 41 | Ovary (54) | Sister – ovary (30's)  Sister – breast (30's)  Mother – pancreas (71)  Maternal first cousin – nose (NR)  Paternal uncle – prostate (NR) |
| POLE c.4411C>T (p.Arg1471Cys) in exon 34 | Breast (43) | Father – liver (40's) |
| POLE c.5278G>A (p.Val1760Met) in exon 39 | Breast (65)  Breast (71) | Sister – breast (40’s) Sister – lung (60’s)  Sister – colon (62)  Sister – kidney (50’s)  Father – pancreas (NR) |
| PTCH1 |  |  |
| PTCH1 c.4025G>A (p.Arg1342His) in exon 23 | Breast (44) | Father – prostate (60’s) Mother – nose (60’s)  Maternal aunt – colon (79)  Maternal aunt –pancreas (60’s) |
| PTCH1 c.212C>T (p.Thr71Ile) in exon 2 | Ovary (49) | Mother's paternal first cousin – breast (50’s) |
| PTCH1 c.1247C>G (p.Thr416Ser) in exon 9 | Ovary (47) | Mother – colon (83) |
| PTCH1 c.37C>G (p.Arg13Gly) in exon 1 | Breast (81) | Brother – lymphoma (40’s) Niece – breast (40’s)  Maternal aunt – endometrium (50’s)  Paternal aunt – colon (60’s)  Paternal aunt – breast (50’s) |
| PTCH1 c.2105C>G (p.Pro702Arg) in exon 14 | Colon (61) | Brother – nose (49)  Mother – thyroid (70)  Maternal aunt – breast (70’s)  Maternal uncle – pancreas (70’s)  Maternal aunt – throat (70’s)  Maternal first cousin –lung (43)  Maternal first cousin – colon (40’s)  Maternal first cousin – stomach (50’s)  Maternal first cousin – liver (30’s)  Paternal first cousin – breast (50’s)  Paternal first cousin – brain (50’s)  Paternal first cousin – prostate (65) |
| PTCH1 c.3575G>T (p.Arg1192Leu) | Ovary (43) | Mother – breast (59)  Maternal aunt – ovary (50’s) |
| PTCH1 c.1128C>G (p.Phe376Leu) in exon 8 | Breast (28) | Brother – lymphoma (18)  Father – lymphoma (62)  Paternal grandfather – prostate (80’s) |
| Supplementary table 2 (continued) |  |  |
| Mutation | **Personal cancer (age at diagnosis)** | **Family history (age at diagnosis)** |
| PTEN c.1-?79+?Dup | Breast (36)  Thyroid (40) | Maternal uncle – bone (60’s)  Maternal aunt – liver (NR) |
| RAD51C |  |  |
| RAD51C c.403T>C (p.Cys135Arg) in exon 2 | Ovary (53) | Sister – breast (50)  Father – colon (58)  Maternal grandmother – nose (70's) |
| RAD51C c.571+5G>A (intronic) in intron 3 | Ovary (59) | Brother – colon cancer (60’s)  Maternal aunt – breast (50’s)  Maternal uncle – colon (50)  Maternal uncle – liver (NR)  Maternal first cousin – colon (30’s)  Father – stomach (61)  Paternal uncle – nose (60’s) |
| RAD51C c.640C>T (p.Arg214Cys) | Ovary (46) | Mother – breast (45) |
| RAD51D |  |  |
| RAD51D c.864C>T (silent) in exon 9 | Breast (54) | Sister – ovary (45) |
| SDHD |  |  |
| SDHD c.335C>T (p.Thr112Ile) in exon 4 | Breast (38) | Nil |
| SMAD4 |  |  |
| SMAD4 c.746_747delAGinsCC(p.Gln249Pro) in exon 6 | Prostate (53) | Father – prostate (75) |
| SMARCA4 |  |  |
| SMARCA4 c.691G>A (p.Gly231Ser) in exon 4 | Endometrium (41) | Father – lymphoma (46)  Maternal grandfather – colon (78) |
| STK11 |  |  |
| STK11 c.1217C>T (p.Ala406Val) in exon 9 | Breast (42) | Mother – breast (45)  Paternal uncle – lung (55) |
| STK11 c.902G>A (p.Arg301Gln) in exon 7 | Ovary (65) | Nil |
| STK11 c.1229C>T (p.Ala410Val) in exon 9 | Breast (44) | Mother – lymphoma (78)  Maternal uncle – prostate (60’s)  Maternal grandmother – breast (50’s)  Maternal grandfather – bladder (NR)  Father – bladder (80)  Paternal aunt – breast (60’s) |
| STK11 c.64A>G (p.Met22Val) | Breast (36)  Thyroid (40) | Maternal uncle – bone (60’s)  Maternal aunt – liver (NR) |
| TP53 |  |  |
| TP53 c.1073A>T (p.Glu358Val) in exon 10 | Gallbladder (59)  Pancreas (66) | Sister – breast (66)  Brother – cholangiocarcinoma (63) |
| TP53 c.736A>T (p.Met246Leu) in exon 7 | Breast (32)  Breast (34)  Ovary (45) | Paternal aunt – kidney (NR) |
| TSC1 |  |  |
| TSC1 c.1528G>A (p.Asp510Asn) in exon 15 | Colon (20) | Father – lung (47) Paternal grandfather – colon (NR) |
| Supplementary table 2 (continued) |  |  |
| Mutation | **Personal cancer (age at diagnosis)** | **Family history (age at diagnosis)** |
| TSC1 c.593A>G (p.Asn198Ser) in exon 7 | Gallbladder (59)  Pancreas (66) | Sister – breast (66)  Brother – cholangiocarcinoma (63) |
| TSC2 |  |  |
| TSC2 c.4420A>G (p.Arg1474Gly) in exon 34 | Ovary (46) | Mother – breast (56, 60), lymphoma (66)  Maternal aunt – nose (50’s)  Maternal uncle – nose (50’s)  Father – colon (76)  Paternal uncle – pancreas/liver (60’s) |
| TSC2 c.2585C>T (p.Ala862Val) in exon 23 | Breast (56) | Mother – larynx (63)  Niece – ovary (65)  Niece – kidney (32) |
| TSC2 c.2225C>T (p.Ser742Leu) in exon 21 | Kidney (39) | Maternal grandfather – colon (70’s), esophagus (90)  Paternal grandmother – lung (70’s) |
| TSC2 c.4346C>T (p.Ser1449Phe) in exon 34 | Breast (38) | Nil |
| TSC2 c.4457C>T (p.Ala1486Val) in exon 34 | Breast (56) | Mother – breast (66), lung (84)  Maternal aunt – cervix (63)  Maternal uncle – leukemia (57)  Maternal first cousin – colon (27)  Father – colon (67)  Paternal uncle – prostate (78)  Paternal grandfather – colon (64) |
| TSC2 c.3070G>A (p.Glu1024Lys) in exon 27 | Ovary (50)  Breast (53)  Peritoneum (69) | Daughter – breast (38)  Sister – breast (48)  Maternal aunt – breast (30) |
| VHL |  |  |
| VHL c.315G>A (silent) in exon 1 | Endometrium (53)  Breast (57)  Breast (69) | Nil |
| VHL c.434A>T (p.Gln145Leu) in exon 2 | Colon (30) | Paternal aunt – colon (60’s) |
| VHL c.3G>T (initiator codon) in exon 1 | Breast (25) | Nil |
